# A lactate metabolism-related gene signature to diagnose osteoarthritis based on machine learning combined with experimental validation

**DOI:** 10.18632/aging.205873

**Published:** 2024-10-16

**Authors:** Jianhua Yang, Wenjun Li, Xuemei Lin, Wei Liang

**Affiliations:** 1 Department of Pain Medicine, Yuebei People’s Hospital, Wujiang, Shaoguan 512000, Guangdong Province, China; 2 Department of Traditional Chinese Orthopedics and Traumatology, Yuebei People’s Hospital, Wujiang, Shaoguan 512000, Guangdong Province, China; 3 Department of Pediatric Orthopedics, Guangzhou Women and Children’s Medical Center, Tianhe, Guangzhou 510623, Guangdong Province, China

**Keywords:** lactate metabolism, osteoarthritis, diagnosis, machine learning

## Abstract

Background: Lactate is gradually proved as the essential regulator in intercellular signal transduction, energy metabolism reprogramming, and histone modification. This study aims to clarify the diagnosis value of lactate metabolism-related genes in osteoarthritis (OA).

Methods: Lactate metabolism-related genes were retrieved from the MSigDB. GSE51588 was downloaded from the Gene Expression Omnibus (GEO) as the training dataset. GSE114007, GSE117999, and GSE82107 datasets were adopted for external validation. Genomic difference detection, protein-protein interaction network analysis, LASSO, SVM-RFE, Boruta, and univariate logistic regression (LR) analyses were used for feature selection. Multivariate LR, Random Forest (RF), Support Vector Machine (SVM), and XGBoost (XGB) were used to develop the multiple-gene diagnosis models. 12 control and 12 OA samples were collected from the local hospital for re-verification. The transfection assays were conducted to explore the regulatory ability of the gene to the apoptosis and vitality of chondrocytes.

Results: Through the bioinformatical analyses and machine learning algorithms, SLC2A1 and NDUFB9 of the 273 lactate metabolism-related genes were identified as the significant diagnosis biomarkers. The LR, RF, SVM, and XGB models performed impressively in the cohorts (AUC > 0.7). The local clinical samples indicated that SLC2A1 and NDUFB9 were both down-regulated in the OA samples (both P < 0.05). The knockdown of NDUFB9 inhibited the viability and promoted the apoptosis of the CHON-001 cells treated with IL-1beta (both P < 0.05).

Conclusions: A lactate metabolism-related gene signature was constructed to diagnose OA, which was validated in multiple independent cohorts, local clinical samples, and cellular functional experiments.

## INTRODUCTION

Osteoarthritis (OA) is the most common orthopedic disorder and is characterized by progressive damage of articular cartilage and its surrounding structures, such as subchondral bone and synovial tissues [1, 2]. The epidemiological study revealed that there are about 300 million patients with hip and/or knee OA, and OA serves as one of the leading causes of disability around the world [3]. To date, plenty of pathological mechanisms have been disclosed, including mechanical stress, immune response, programmed cell death, and energy metabolism, providing therapeutic opportunities [4–7]. Based on these efforts, some clinical trials associated with the therapy strategies targeting the disease-causing genes, including TGF-beta, IFN-beta, IL10, IL-1R, and NKX3, have been performed [8]. These bracing advancements enlighten the researchers to seek more gene therapy targets, which is the aim of this study as well.

Lactate was considered the waste substance of anaerobic glycolysis in the past. However, emerging evidence has disclosed its pivotal biological functions and regulatory ability in different cellular processes [9]. Lactate was able to regulate adipocyte differentiation [10], tumor immune escape [11], and neuronal network activity [12] by interacting with its receptor GPR81. Histones are vital components in the chromosome, and their epigenetic modification plays important roles in the regulation of diverse cellular activities. Lactylation is a newly discovered histone modification type and has been proven as the regulator in M2 macrophage polarization, cell metabolic processes, and so forth [13]. The tight association between lactate and OA has also been reported. Compared with the normal hip, the concentration of lactate exhibited a 50% increase in the OA hip [14]. Hurter et al. found that the levels of lactate dehydrogenase in the synovial fluid could act as a potential diagnostic biomarker for OA [15]. Nevertheless, the number of studies focusing on the interaction between lactate metabolism and the pathogenesis of OA is still limited currently. Comprehensive analyses of lactate metabolism-related genes to unearth novel biomarkers of OA and uncover the underlying pathological mechanisms are urgently demanded.

Herein, the present study aims to develop a lactate metabolism-related gene signature as the diagnosis biomarker in OA. Diverse machine learning algorithms, including least absolute shrinkage and selection operator (LASSO), Boruta, supporter vector machine-recursive feature elimination (SVM-RFE), logistic regression (LR), random forest (RF), supporter vector machine (SVM), and XGBoost (XGB) were used for feature selection and model construction. 24 clinical samples collected from the local hospital were utilized to re-confirm our findings. The transfection assays were conducted to elucidate the regulatory relationship between the identified gene and the proliferation and apoptosis of chondrocytes.

## MATERIALS AND METHODS

### Data collection

273 lactate metabolism-related genes were retrieved from the Molecular Signatures Database (MSigDB, http://www.gsea-msigdb.org/gsea/msigdb/index.jsp), as displayed in Supplementary Table 1. GSE51588 dataset [16], which included the transcriptome sequencing data of the subchondral bone tissues isolated from 10 control and 40 OA patients, was obtained from the Gene Expression Omnibus (GEO, https://ncbi.nlm.nih.gov/geo/) as the training cohort. GSE114007 [17], GSE117999, and GSE82107 [18] datasets, which were also downloaded from the GEO, were selected for external validation. It should be stated that GSE114007 and GSE117999 datasets contained the transcriptome sequencing of the cartilage tissues, while the GSE82107 dataset included the transcriptome sequencing of synovial tissue. Therefore, GSE114007 and GSE117999 datasets were merged as one dataset, namely “Validation-cartilage” cohort. GSE82107 dataset was called “Validation-synovial” in this study. To further detect the ability of the genes to distinguish OA from rheumatoid arthritis (RA), GSE89408 dataset [19], which included the transcription sequencing data of the joint synovial biopsies from 22 OA and 152 RA samples, was also downloaded from GEO and named as “OA vs. RA” cohort. The batch effects across these experiments were reduced via the sva package in R as possible. More detailed information on these public cohorts can be seen in Supplementary Table 2.

### Gene expression difference detection and protein-protein interaction (PPI) network construction

The differentially-expressed genes between the control and OA samples were identified via the limma package in R with |log fold change [FC]| > 0.5 and false discovery rate (FDR) < 0.05 filtering. Subsequently, the lactate metabolism-related genes showing expression differences were included in the PPI network based on the STRING database (https://cn.string-db.org/) with a confidence level = 0.4. The cytoHubba app in the Cytoscape (version 3.8.0) was harnessed to quantify the importance of the gene in the PPI network, where the degree algorithm was adopted. The top 25 genes exhibiting the highest degree were selected for further analysis.

### Functional enrichment and gene set enrichment analysis (GSEA)

The functional annotation of the differentially-expressed genes was performed through the ClueGo plug-in in the Cytoscape software and the Metascape database (https://metascape.org/gp/index.html#/main/step1). The Gene Ontology (GO) terms with P < 0.05 were displayed in the network. GSEA was conducted through the GSEA software (version 4.3.2), and the Hallmark signatures, which were downloaded from the MSigDB, were chosen as the reference datasets. The signatures with nominal P < 0.05 and FDR < 0.05 were considered to be statistically significant.

### Feature selection

Multiple machine learning algorithms were simultaneously adopted to identify the genes as the significant diagnosis biomarkers of OA. LASSO regression with 10-fold cross-validation was implemented via the glmnet package. The caret package in R was utilized to carry out the SVM-RFE algorithm. Boruta algorithm rendered each variable labeled “Tentative,” “Rejected,” or “Confirmed,” which represented the influence of the variable on the outcomes [20], and the Boruta package in R was used for the algorithm implementation. Univariate LR was performed to clarify the diagnosis value of the variables using the rms package, and P < 0.01 was set as the filtering threshold. At last, the genes co-determined by the PPI network analysis, LASSO, SVM-RFE, Boruta, and univariate LR were identified as the significant diagnosis biomarkers of OA and then included in the diagnosis models.

### Unsupervised clustering

The consensus clustering was performed to divide the cartilage samples into different clusters using the ConsensusClusterPlus package in R software, and then the clustering was validated through the Principal Component Analysis (PCA) by the “prcomp” function in R. The differentially-expressed genes among the clusters were detected by the limma package, and |logFC| > 2 and FDR < 0.05 were set as the filtering thresholds.

### Diagnosis model construction and validation

Based on the identified genes, we used multiple algorithms, including multivariate LR, RF, SVM, and XGB, to develop the diagnosis models using the train function of the caret package. A nomogram was drawn to visualize the LR model via the rms package. We also utilized the calibrate function of the rms package to conduct the calibration analysis. The pROC package was used to draw the receiver operating characteristic (ROC) curves to quantify the performance of the models in the training and external validation cohorts. Decision curve analysis (DCA) was performed to clarify the predictive ability of the models through the dcurves package of R.

### Meta-analyses

Meta-analyses were conducted to pool the odds ratios (ORs) to better clarify the diagnosis value using the meta package in R. The fixed- or random-effects model would be adopted according to the results of heterogeneity test.

### Clinical sample collection

The knee cartilage tissues extracted from 12 subjects undergoing traumatic amputation without rheumatoid arthritis or OA and 12 OA subjects going through total knee replacement were obtained from the Yuebei People’s Hospital between 2021 and 2023. The Ethics Committee of Yuebei People’s Hospital reviewed and approved this research project according to the principles of the Declaration of Helsinki. The knee cartilage samples were stored in liquid nitrogen for RNA isolation. These samples collected from our hospital were included in “Validation-local” cohort in this study. The baseline clinicopathological features of these subjects can be found in Supplementary Table 3.

### Enzyme-linked immunosorbent assay (ELISA)

The levels of lactate in the knee cartilage tissues from the control and OA subjects were measured by the L-lactate assay kit (Abcam, The Netherlands) following the manufacturer’s instructions. As previously mentioned [21], the samples were treated with lactate assay buffer (Abcam, The Netherlands) and the endogenous lactate dehydrogenase was removed using the deproteinizing sample preparation kit-TCA (Abcam, The Netherlands). The lactate concentration of the samples was then determined using a microplate reader.

### Cell culture and treatment

Human immortalized chondrocyte CHON-001 cell line was purchased from the American Type Culture Collection (USA) and maintained in RPMI-1640 medium (Thermo Fisher Scientific, USA) supplemented with 10% FBS and 1% penicillin-streptomycin at 37° C in a humidified atmosphere with 5% CO2. 10 ng/mL IL-1β (Sigma-Aldrich, China) was used to treat the CHON-001 cells for 48 hours to mimic OA, as previously described [22, 23].

The transient transfection assays in the CHON-001 cells were conducted following the manufacturer’s protocol of Lipofectamine 2000 reagent (Invitrogen, USA). The small interfering RNA (siRNA) targeting NDUFB9 and the negative control siRNA (NC) were designed and synthesized by the Biosyntech company (Suzhou, China). The siRNA sequences can be found in Supplementary Table 4. Real-time quantitative PCR (RT-qPCR) experiments were used to measure the knockdown efficacy of the siRNAs.

### RT-qPCR

The total RNA isolation of the CHON-001 cells and human cartilage tissues was conducted using the TRIzol reagent (Thermo Fisher Scientific, USA). Transcriptor First Strand cDNA Synthesis Kit (Roche, Switzerland) was used for the synthesis of cDNA, and the SYBR Premix ExTaq kit (TaKaRa, China) was implemented for the RT-qPCR experiments based on the ABI 7500 system (Life Technology, USA). GAPDH was chosen as the reference gene, and the results were normalized via the 2^-ΔΔCt^. The primer sequence in these RT-qPCR experiments is shown in Supplementary Table 5.

### Cell viability and apoptosis

The cell viability of CHON-001 cells was assessed with the Cell Counting Kit-8 (CCK-8, Sigma-Aldrich, USA) on a 96-well plate at a density of 1×10^5^ cells/well. After the treatment, 10 μl CCK-8 reagent was added to the platelets and then co-incubated with the cells for 2 hours at 37° C. The optimal density (OD) value of each well was measured by a microplate reader (Bio-Rad, USA) at an absorbance of 450 nm.

The apoptosis levels of the CHON-001 cells treated with IL-1beta were measured with the Hoechst 33342 reagent (Invitrogen, USA) according to the manufacturer’s suggestions. The images were then taken by fluorescence microscopy. The ImageJ software (version 1.54d) was used to analyze the fluorescence intensity to quantify the apoptosis levels. Additionally, to further verify our finding, the flow cytometry apoptosis assay was also conducted. The flow cytometry apoptosis analysis was conducted using a fluorescein isothiocyanate-conjugated annexin V apoptosis detection kit I (BD Biosciences, USA) following the manufacturer’s instructions. The analysis of the apoptosis levels of these samples was based on a flow cytometry system (BD Biosciences, USA).

### Statistical analyses

The statistical analyses of the whole study were based on the R software (version 4.2.0). Welch’s corrected t-test was applied to compare the difference of the values detected by the RT-qPCR, CCK-8, and apoptosis staining. Unless otherwise specified, P < 0.05 was accepted as the significance threshold. *P < 0.05; **P < 0.01; ***P < 0.001.

### Data availability

The data that support the findings of this study are openly available in GEO (https://www.ncbi.nlm.nih.gov/geo/) and MSigDB (https://www.gsea-msigdb.org/gsea/msigdb/index.jsp). The R codes used in this study can be obtained from the corresponding author upon reasonable request.

## RESULTS

### 74 of 273 lactate metabolism-related genes were differentially expressed

**Figure 1:**
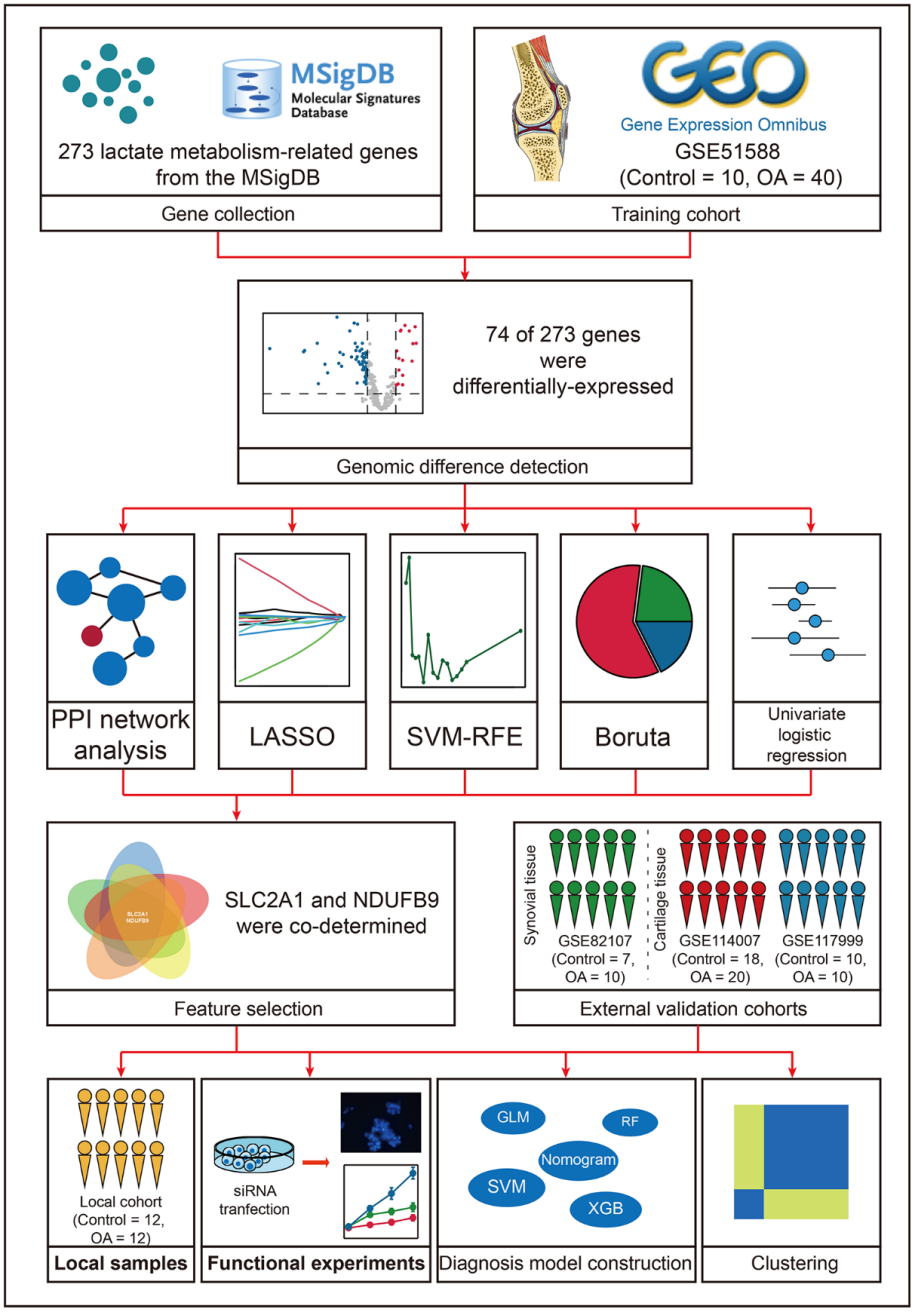
The workflow of the present study.

**Figure 2:**
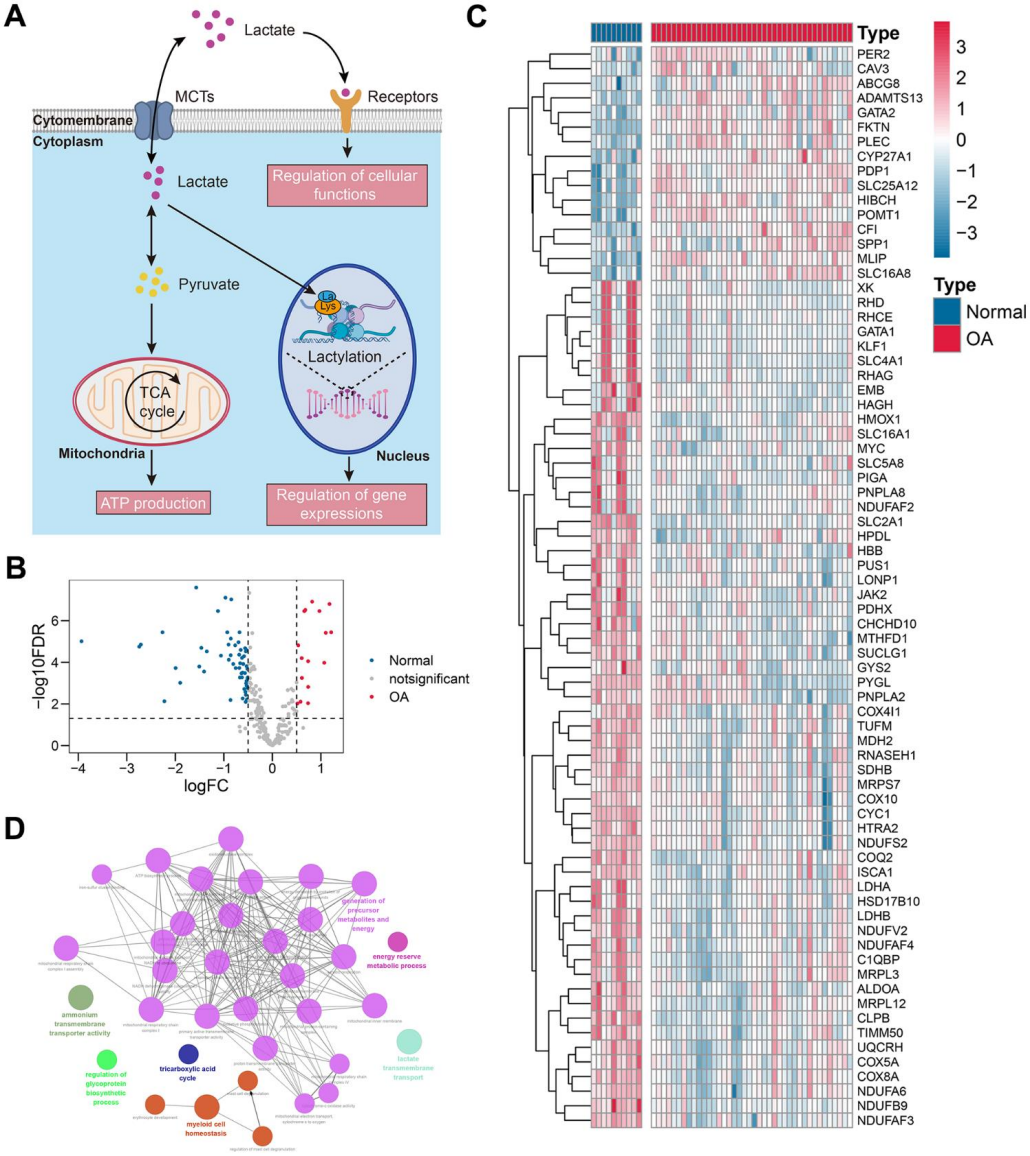
**Differentially-expressed lactate metabolism-related genes.** (**A**) The schematic summary of the biological functions of lactate. (**B**, **C**) The volcano plot (**B**) and the heat map (**C**) indicated that 74 of 273 lactate metabolism-related genes were differentially expressed between the control and OA tissues. (**D**) The functional annotation of the 74 genes. Abbreviation: OA, osteoarthritis.

Figure 1 graphically describes the workflow of this study. First, we evaluated the lactate levels in the knee cartilage tissues from the control and OA subjects via ELISA assay. The results indicated that the levels of lactate in the OA samples were higher than those in control cases (P < 0.05, Supplementary Figure 1), implying that lactate might exert important functions in the pathogenesis of OA. As stated above, increasing evidence has uncovered that lactate exerts important functions in energy metabolism, signal transduction, and lactylation (Figure 2A), enlightening us to investigate the roles of lactate metabolism-related genes in OA. Based on the GSE51588 cohort, a sum of 74 of 273 lactate metabolism-related genes were differentially expressed between the control and OA samples (Figure 2B and Supplementary Table 6). The expression levels of these 74 genes are visualized in the heatmap (Figure 2C). The GO enrichment analysis indicated that these genes were mainly involved in the energy metabolic process, glycoprotein synthesis, lactate transport, and cell homeostasis maintenance (Figure 2D), indicating the underlying functions of lactate metabolism-related genes in the pathogenesis of OA.

### SLC2A1 and NDUFB9 were identified as significant diagnosis biomarkers in OA

**Figure 3:**
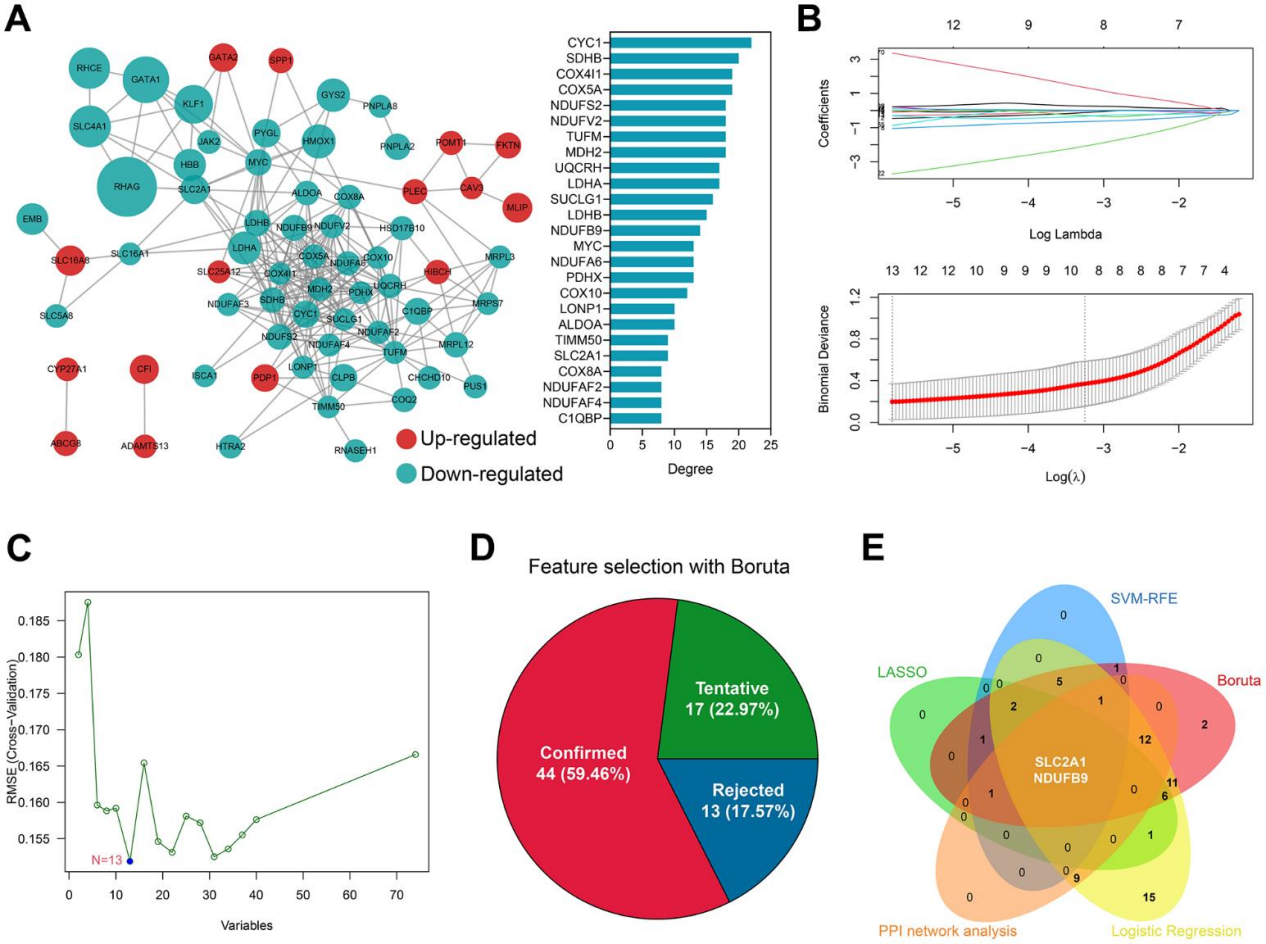
**SLC2A1 and NDUFB9 were co-determined by bioinformatical analyses and machine learning algorithms.** (**A**) The construction and analyses of the PPI network. (**B**) 13 genes were identified by the LASSO regression. (**C**) 13 genes were determined by the SVM-RFE algorithm. (**D**) Boruta algorithm showed that 44 genes were of high diagnosis value. (**E**) SLC2A1 and NDUFB9 were co-determined by the LASSO, SVM-RFE, Boruta, univariate logistic regression, and PPI network analysis. Abbreviations: LASSO, least absolute shrinkage and selection operator; SVM-RFE, supporter vector machine-recursive feature elimination; PPI, protein-protein interaction.

A PPI network was constructed to investigate the underlying interactions of the 74 differentially-expressed genes, and the Top 25 genes sharing the highest degree in the network were chosen for further analysis (Figure 3A). 13 genes were identified by the LASSO regression, including XK, GATA1, HMOX1, SLC2A1, CYC1, HAGH, NDUFB9, HTRA2, ISCA1, HIBCH, FKTN, PDP1, and SLC16A8 (Figure 3B and Supplementary Table 7). Meanwhile, the SVM-RFE algorithm determined 13 genes as significant factors (Figure 3C and Supplementary Table 8), The Boruta algorithm helped to identify 44 genes (Figure 3D and Supplementary Table 9), and univariate LR analysis indicated that 64 genes were of high diagnosis value (P < 0.01, Supplementary Table 10). Taken together, SLC2A1 and NDUFB9 were co-determined by the PPI network analysis, LASSO, SVM-RFE, Boruta, and univariate LR (Figure 3E), according to which the diagnosis models were constructed.

### The unsupervised clustering based on the expressions of SLC2A1 and NDUFB9

**Figure 4:**
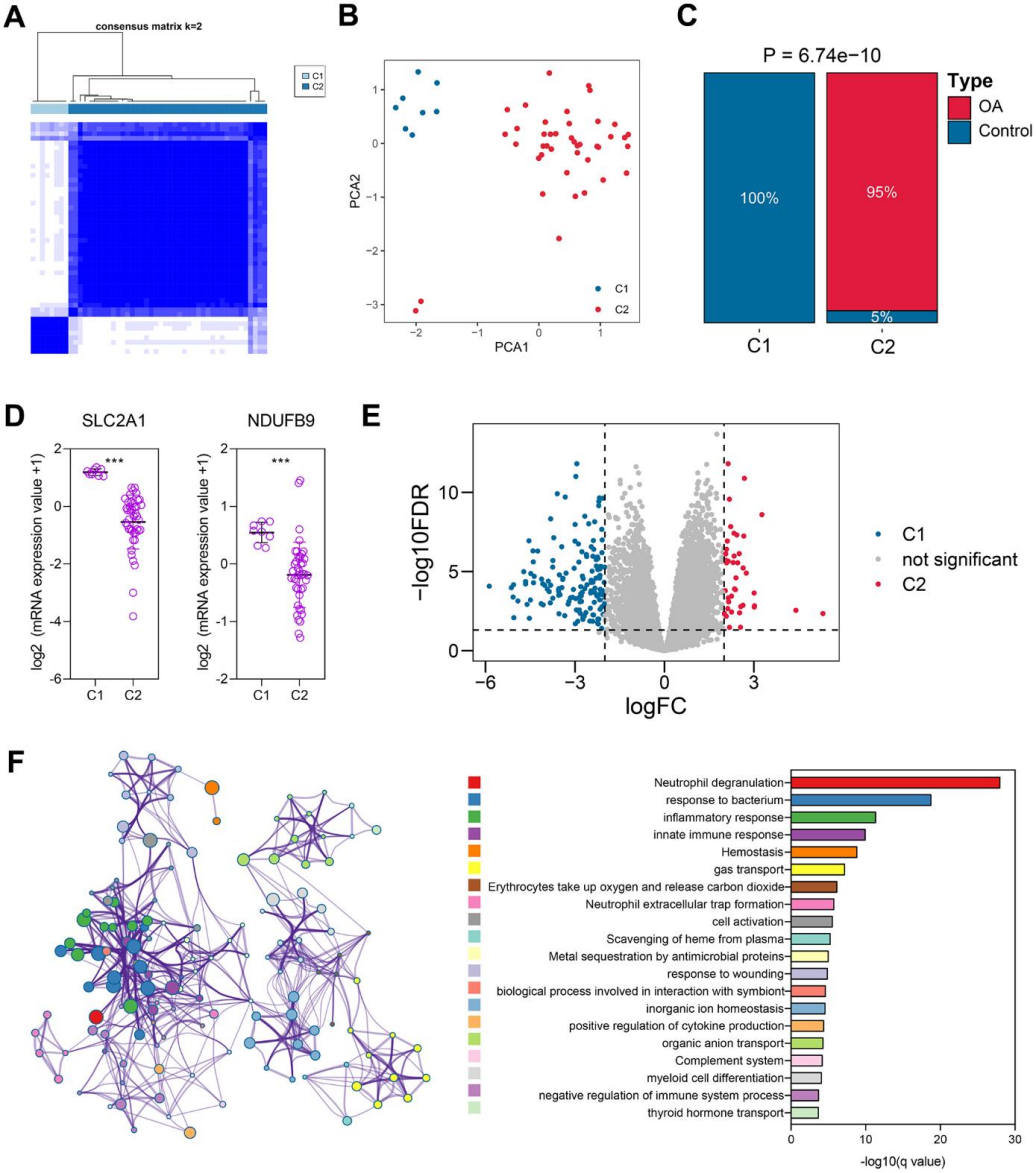
**The unsupervised clustering based on the expressions of NDUFB9 and SLC2A1.** (**A**) 50 cartilage samples were divided into two different clusters using the consensus clustering algorithm. (**B**) Principal Component Analysis was performed to confirm the reliability of the clustering. (**C**) The clustering was associated with OA features. (**D**) The expressions of NDUFB9 and SLC2A1 were significantly altered in these two clusters. (**E**) 190 genes, which were differentially expressed between cluster 1 and cluster 2, were identified. (**F**) The functional enrichment of the 190 genes. ***P < 0.001.

According to the expressions of SLC2A1 and NDUFB9, 50 cartilage samples were grouped into Cluster 1 (C1) and Cluster 2 (C2), as displayed in Figure 4A and Supplementary Table 11. PCA indicated the distinct genomic patterns of these two clusters (Figure 4B). Importantly, we noticed that about 95% C2 cases belong to OA subjects, and none of the C1 cases exhibited OA phenotype (P < 0.001, Figure 4C), suggesting that the clustering was associated with the OA characteristics. The expressions of SLC2A1 (P < 0.001) and NDUFB9 (P < 0.001) were significantly down-regulated in C2 subjects (Figure 4D). Subsequently, the differentially-expressed genes between the C1 and C2 subjects were analyzed, and a sum of 190 genes were screened (Figure 4E and Supplementary Table 12). The functional enrichment displayed that these genes were mainly associated with the cell activation and differentiation, immune and inflammatory response, and metabolic processes (Figure 4F), implying the possible functions of SLC2A1 and NDUFB9.

### SLC2A1 and NDUFB9 were reliable diagnosis biomarkers in the meta-analyses

**Figure 5:**
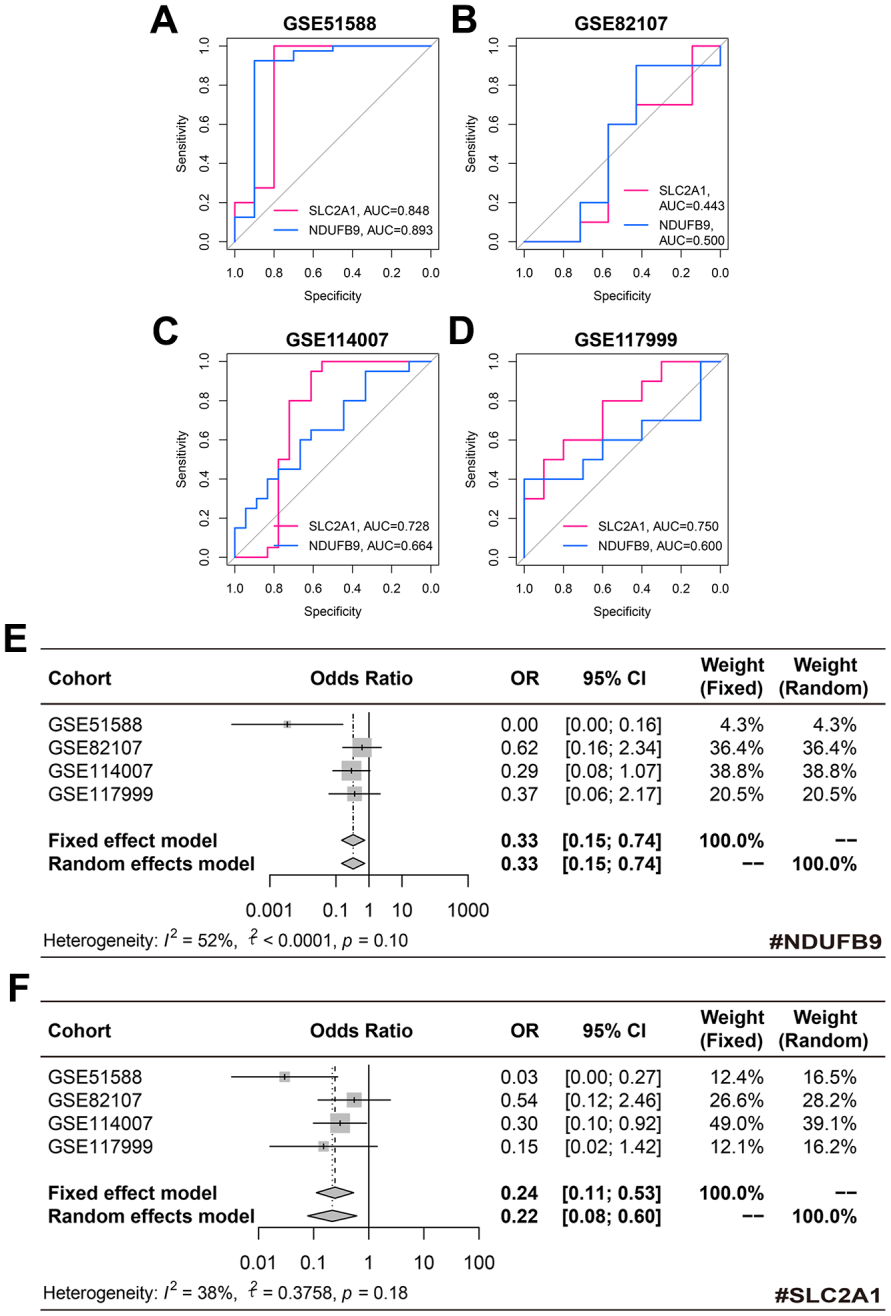
**The diagnosis value of NDUFB9 and SLC2A1.** (**A**–**D**) The diagnostic performance of NDUFB9 and SLC2A1 in the training (**A**), GSE82107 (**B**), GSE114007 (**C**), and GSE117999 (**D**) cohorts. (**E**, **F**) The meta-analyses were used to pool the ORs of NDUFB9 (**E**) and SLC2A1 (**F**). Abbreviation: OR, odds ratio.

The diagnostic performance of SLC2A1 and NDUFB9 in the training, GSE82107, GSE114007, and GSE117999 cohorts was displayed in Figure 5A–5D, respectively. Despite the fact that these genes performed poorly in some cohorts, the meta-analyses indicated that NDUFB9 (pooled OR = 0.33, 95% confidence interval [CI] = 0.15-0.74, Figure 5E) and SLC2A1 (pooled OR = 0.24, 95% CI = 0.11-0.53, Figure 5F) both served as significant diagnosis biomarkers in OA subjects.

### The performance of the machine learning-based diagnosis models

**Figure 6:**
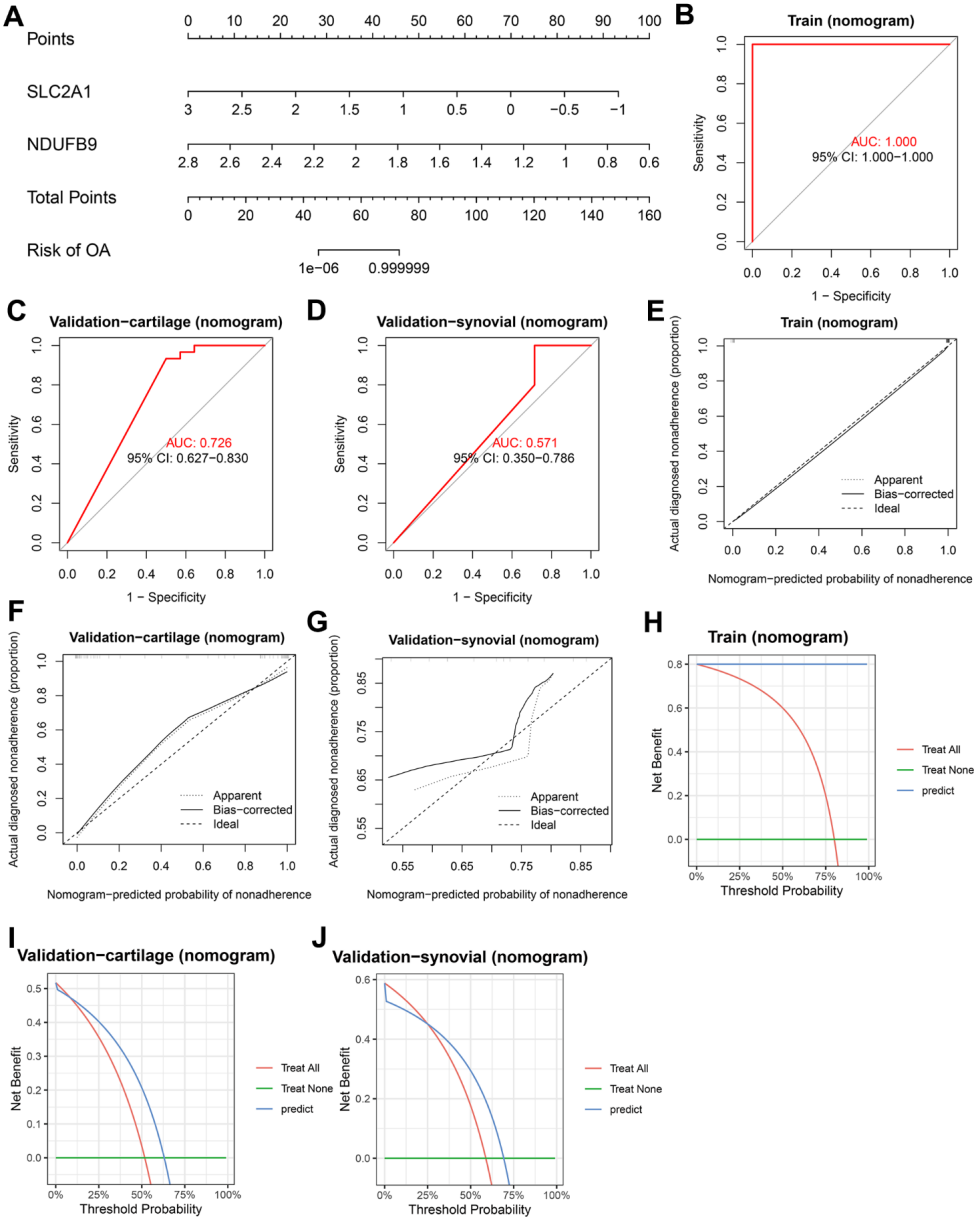
**Construction and validation of the diagnostic nomogram.** (**A**) A nomogram was constructed to visualize the LR model. (**B**–**D**) ROC analysis shows the predictive ability of the nomogram in the training (**B**), validation-cartilage (**C**), and validation-synovial (**D**) cohorts. (**E**–**G**) Calibration analysis shows the predictive ability of the nomogram in the training (**E**), validation-cartilage (**F**), and validation-synovial (**G**) cohorts. (**H**–**J**) DCA analysis shows the predictive ability of the nomogram in the training (**H**), validation-cartilage (**I**), and validation-synovial (**J**) cohorts. Abbreviations: ROC, receiver operating characteristic; DCA, decision curve analysis; LR, logistic regression.

Multiple-gene diagnosis models were developed using LR, RF, SVM, and XGB based on the mRNA expression levels of SLC2A1 and NDUFB9. First, a nomogram was constructed using the established LR model (Figure 6A). ROC analyses were performed to evaluate the predictive performance of the nomogram in the training (area under curve [AUC] = 1.000, 95%CI = 1.000-1.000, Figure 6B), validation-cartilage (AUC = 0.726, 95%CI = 0.627-0.830, Figure 6C), and validation-synovial (AUC = 0.571, 95%CI = 0.350-0.786, Figure 6D) cohorts. The calibration plots demonstrated that the nomogram had acceptable predictive capability in the training (Figure 6E), validation-cartilage (Figure 6F), and validation-synovial (Figure 6G) cohorts. Furthermore, decision curve analysis (DCA) was conducted to assess the net benefit of the nomogram at different decision thresholds in these cohorts (Figure 6H–6J).

**Figure 7:**
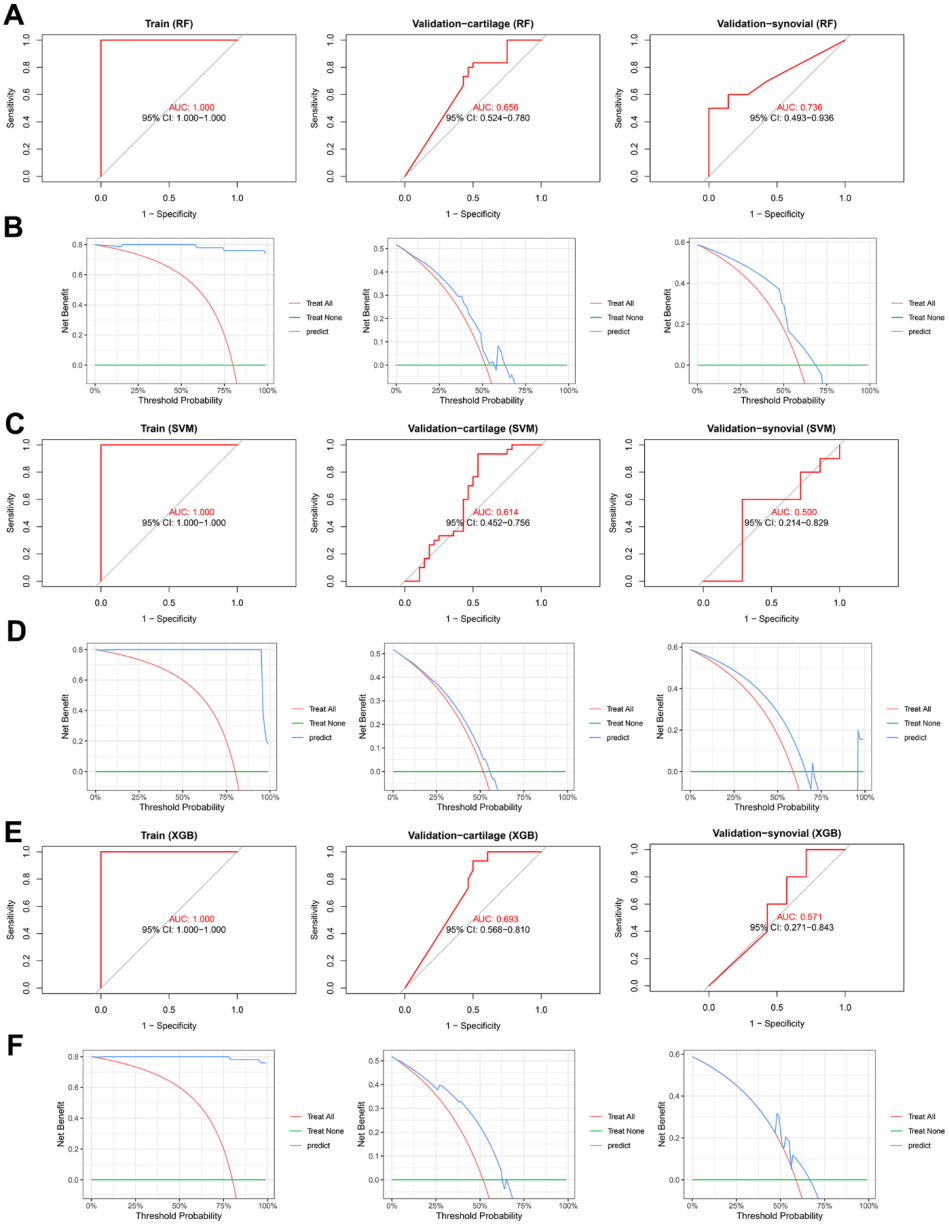
**The predictive performance of the RF, SVM, and XGB diagnosis models.** (**A**, **B**) ROC (**A**) and DCA (**B**) analysis indicates the predictive performance of RF model in the training (left), validation-cartilage (middle), and validation-synovial (right) cohorts. (**C**, **D**) ROC (**C**) and DCA (**D**) analysis indicates the predictive performance of SVM model in the training (left), validation-cartilage (middle), and validation-synovial (right) cohorts. (**E**, **F**) ROC (**E**) and DCA (**F**) analysis indicates the predictive performance of XGB model in the training (left), validation-cartilage (middle), and validation-synovial (right) cohorts. Abbreviations: RF, random forest; SVM, supporter vector machine; XGB, XGBoost; AUC, area under curve; CI, confidence interval.

In addition to the nomogram, alternative machine learning-based models were also developed, as previously mentioned. ROC analyses demonstrated that the RF model exhibited AUCs of 1.000 (95%CI = 1.000-1.000), 0.656 (95% CI = 0.524-0.780), and 0.736 (95%CI = 0.493-0.936) in the training, validation-cartilage, and validation-synovial cohorts, respectively (Figure 7A). Furthermore, the DCA showcased the net benefit of the RF model at various thresholds in these cohorts (Figure 7B). Likewise, the SVM diagnostic model demonstrated AUCs of 1.000 (95%CI = 1.000-1.000), 0.614 (95%CI = 0.452-0.756), and 0.500 (95%CI = 0.214-0.829) in the training, validation-cartilage, and validation-synovial cohorts (Figure 7C). DCA was also employed to further elucidate the predictive capability of the SVM model in these datasets (Figure 7D). Similarly, the XGB model exhibited impressive performance in the training (AUC = 1.000, 95%CI = 1.000-1.000, Figure 7E), validation-cartilage (AUC = 0.693, 95%CI = 0.568-0.810, Figure 7E), and validation-synovial (AUC = 0.571, 95%CI = 0.271-0.843, Figure 7E) cohorts. The results of DCA for the XGB model in these cohorts are illustrated in Figure 7F. Additionally, the calibration analyses of the RF (Supplementary Figure 2A), SVM (Supplementary Figure 2B), and XGB (Supplementary Figure 2C) in the training, validation-cartilage, and validation-synovial cohorts were also performed. Overall, despite the suboptimal performance of these models in certain cohorts, we have established that the gene signature, encompassing SLC2A1 and NDUFB9, represents a potential diagnostic biomarker for OA, irrespective of whether it is present in cartilage or synovial tissues.

### Validation in the local cohort

**Figure 8:**
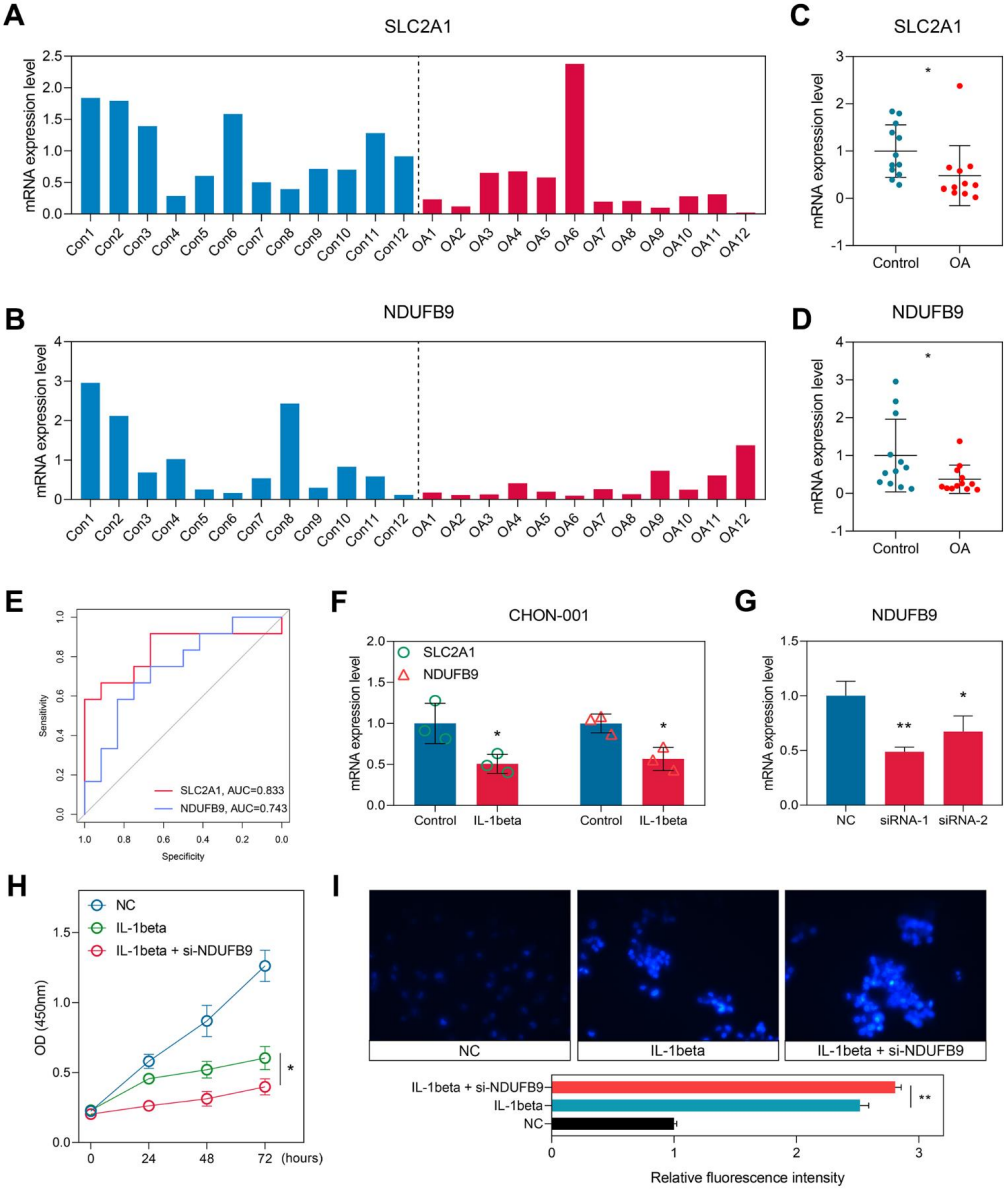
**Experimental validation in the clinical samples and cellular experiments.** (**A**, **B**) The expression levels of SLC2A1 (**A**) and NDUFB9 (**B**) in the 12 control and 12 OA samples collected from the local hospital. (**C**, **D**) The levels of SLC2A1 (**C**) and NDUFB9 (**D**) were down-regulated in the OA tissues. (**E**) The diagnostic performance of SLC2A1 and NDUFB9 in the local cohort. (**F**) SLC2A1 and NDUFB9 were both down-regulated in the CHON-001 cells treated with IL-1beta. (**G**) Two different siRNAs were used to construct the NDUFB9-knockdown CHON-001 cells. (**H**) The proliferation rate was inhibited in the NDUFB9-knockdown CHON-001 cells. (**I**) The CHON-001 cells with the knockdown of NDUFB9 showed higher levels of apoptosis after the IL-1beta treatment. *P < 0.05; **P < 0.01.

To further confirm our findings, we collected the cartilage tissues isolated from 12 control and 12 OA subjects in our local hospital. RT-qPCR experiments were conducted to measure the expression levels of SLC2A1 (Figure 8A) and NDUFB9 (Figure 8B), and the results indicated that SLC2A1 (P < 0.05, Figure 8C) and NDUFB9 (P < 0.05, Figure 8D) were both down-regulated in the OA samples. The ROC analyses displayed the diagnosis performance of SLC2A1 (AUC = 0.833, 95%CI = 0.639-0.979) and NDUFB9 (AUC = 0.743, 95% CI = 0.528-0.917) in the local cohort (Figure 8E).

Furthermore, in order to provide further insights into the diagnostic capabilities of the established models, we conducted an analysis to assess the predictive performance of the nomogram, RF, SVM, and XGB models in the local cohort. The AUCs of the nomogram, RF, SVM, and XGB models were found to be 0.667 (95%CI = 0.542-0.792, Supplementary Figure 3A), 0.715 (95%CI = 0.510-0.889, Supplementary Figure 3B), 0.580 (95%CI = 0.333-0.826, Supplementary Figure 3C), and 0.674 (95%CI = 0.486-0.837, Supplementary Figure 3D), respectively. Additionally, DCA plots were generated to evaluate the performance of the nomogram, RF, SVM, and XGB models in the local cohort (Supplementary Figure 3E–3H). Moreover, calibration plots were constructed to demonstrate the predictive ability of these models in the local cohort (Supplementary Figure 3I–3L). It is worth noting that the training cohort utilized transcriptome sequencing technology, while the local cohort relied on RT-qPCR experiments. The discrepancy arising from the utilization of distinct gene expression detection platforms could potentially explain the suboptimal performance of the predictive models in the local cohort.

### Knockdown of NDUFB9 inhibited the proliferation and promoted the apoptosis of cartilage cells

Human Immortalized chondrocyte CHON-001 cells were treated with IL-1beta to construct the OA cell model. We observed that the expressions of SLC2A1 and NDUFB9 were both down-regulated in the OA cell models (both P < 0.05, Figure 8F). Since the roles of SLC2A1 in OA have been reported by previous efforts [24, 25], we then selected NDUFB9 to conduct the functional investigation. Two different siRNAs targeting NDUFB9 were used for the gene knockdown, and siRNA-2 exhibited higher efficacy (P < 0.01, Figure 8G) and was chosen for further analysis. The knockdown of NDUFB9 significantly suppressed the proliferation (P < 0.05, Figure 8H) and enhanced the apoptosis (P < 0.05, Figure 8I) of the CHON-001 cells treated with IL-1beta. In order to gain further insights into the impact of NDUFB9 on the IL-1beta-induced apoptosis of CHON-001 cells, we conducted additional flow cytometry apoptosis analysis. The results obtained from the flow cytometry assay provide additional validation for our findings (P < 0.05, Supplementary Figure 4).

### The signal pathways associated with SLC2A1 and NDUFB9 in OA

**Figure 9:**
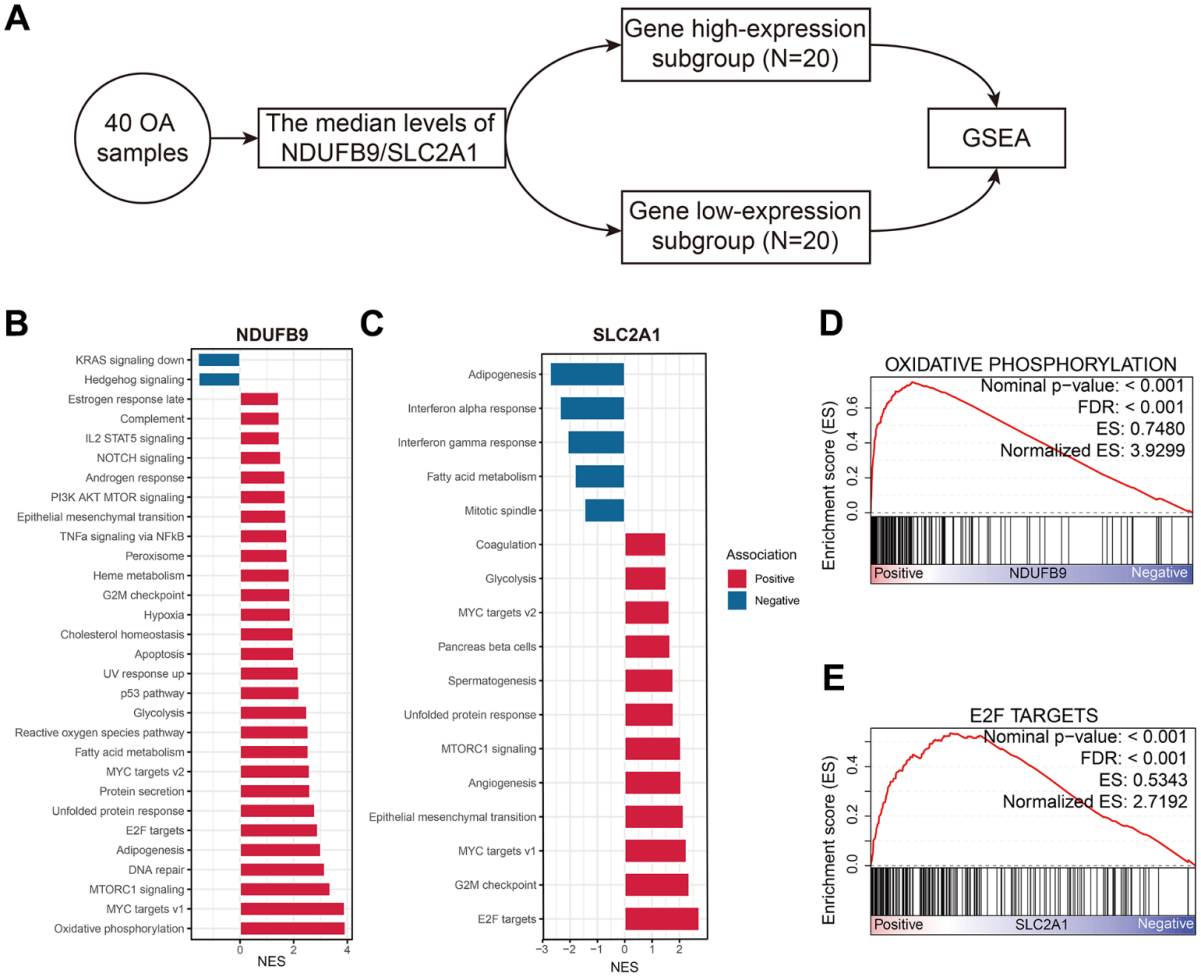
**The functional annotation of NDUFB9 and SLC2A1 in the OA samples.** (**A**) GSEA was conducted according to the median expression levels of the genes. (**B**, **C**) The biological processes associated with NDUFB9 (**B**) and SLC2A1 (**C**). (**D**, **E**) The signal pathways most relevant to NDUFB9 (**D**) and SLC2A1 (**E**). Abbreviation: GSEA, Gene Set Enrichment Analysis.

The OA samples extracted from the GSE51588 cohort were equally divided into the low- and high-gene expression subgroups, and then the GSEA was conducted (Figure 9A). The signal pathways associated with NDUFB9 and SLC2A1 were shown in Figure 9B and Figure 9C, respectively. The most relevant pathways of NDUFB9 and SLC2A1 are oxidative phosphorylation (NES= 3.930, FDR < 0.001, Figure 9D) and E2F targets (NES = 2.719, FDR < 0.001, Figure 9E), which indicated the underlying mechanisms of these genes in the pathogenesis of OA.

### The ability of SLC2A1 and NDUFB9 to distinguish OA from RA

OA and RA are both prevalent joint disorders, characterized by overlapping clinical manifestations including joint pain, swelling, stiffness, and morning stiffness. These shared symptoms pose a challenge in accurately diagnosing these conditions in clinical settings. Consequently, we aimed to investigate the discriminative potential of SLC2A1 and NDUFB9 in distinguishing between OA and RA, utilizing a sample cohort of 22 OA and 152 RA samples obtained from the GSE82107 dataset. Upon analysis, it was observed that RA tissues exhibited significantly higher levels of NDUFB9 compared to OA samples (P < 0.05, Supplementary Figure 5A), while no significant expression difference was observed for SLC2A1 (P > 0.05, Supplementary Figure 5A). The area under the curve (AUC) values for NDUFB9 and SLC2A1 were calculated as 0.648 (95%CI = 0.510-0.781, Supplementary Figure 5B) and 0.580 (95%CI = 0.418-0.728, Supplementary Figure 5C), respectively. These findings suggest that NDUFB9 may serve as a potential biomarker for distinguishing between OA and RA.

## DISCUSSION

Seeking novel biomarkers in OA has always been a hot topic, which helps to perform the early diagnosis, disclose new pathogenesis, and develop novel targeted drugs. Recently, tremendous advancements have been made in OA biomarker identification with the proposal and application of machine learning algorithms, multi-omics sequencing, and big-data mining [26–28]. Detection of the biomarkers in OA from particular biological aspects, such as immunity [29], cell death [30, 31], RNA-binding proteins [32], and epigenetic regulators [33], is becoming more and more popular. These great efforts expanded our knowledge of the initiation and development of OA and provided the potential tools in clinical practice. However, despite the fact the multiple and pivotal functions of lactate have attracted increasing attention in recent years [34, 35], as discussed above, no comprehensive analysis of the lactate metabolism-related genes as the biomarkers in OA has been performed up to date.

In this study, initially, a total of 12 control and 12 OA samples were collected from our hospital, and the levels of lactate in these tissues were quantified using the ELISA. It was observed that the concentration of lactate in the OA samples was significantly higher than that in the control samples (P < 0.05). Subsequently, a comprehensive analysis was conducted using 173 lactate metabolism-related genes obtained from the MSigDB database, and the GSE51588 dataset from the GEO database was selected as the training cohort. Out of the 273 lactate metabolism-related genes, 74 exhibited differential expression between the control and OA samples. Through the integration of the PPI network, LASSO, SVM-RFE, Boruta, and univariate LR, SLC2A1 and NDUFB9 were identified as significant diagnostic biomarkers. The diagnostic ability of SLC2A1 and NDUFB9 in OA was further validated through unsupervised clustering and meta-analyses. Furthermore, multiple machine learning diagnosis models, including LR, RF, SVM, and XGB, were constructed based on the expressions of SLC2A1 and NDUFB9. A nomogram was developed to visualize the LR predictive model. These models demonstrated satisfactory performance across the training, validation-cartilage, and validation-synovial cohorts. Notably, the diagnosis models also exhibited effectiveness in synovial tissues, despite being trained on subchondral bone tissues, indicating the widespread involvement of SLC2A1 and NDUFB9 in the pathogenic processes of OA. Subsequently, an independent set of 12 control and 12 OA samples obtained from a local hospital were utilized to confirm our findings (both P < 0.05). Cellular functional experiments were conducted, revealing that the knockdown of NDUFB9 significantly suppressed the viability and enhanced the apoptosis of CHON-001 cells treated with IL-1beta (both P < 0.05). Finally, the ability of SLC2A1 and NDUFB9 to distinguish between OA and RA was evaluated, and the results suggested that NDUFB9 holds promise as a potential biomarker for distinguishing OA from RA.

In this study, we integrated sequencing data from subchondral bone, cartilage, and synovial tissue of OA patients, considering the tight relationship of these three tissues in the pathogenesis of OA [36]. The main pathology of OA is the degeneration of articular cartilage, where the cartilage surface becomes rough, with cracks and wear. After cartilage degeneration, the underlying bone experiences additional pressure and friction, leading to increased bone cell proliferation and bone density, eventually resulting in bone sclerosis and osteophyte formation. Osteophytes can further damage the synovium and cartilage, exacerbating the condition of OA. The degradation and destruction of cartilage release certain cellular factors and inflammatory mediators that can stimulate synovial cells to produce an inflammatory response. Synovitis not only causes synovial lesions but also worsens joint destruction. Inflammatory mediators produced by synovitis, such as cytokines and enzymes, can directly affect the metabolism and degeneration process of cartilage, while also stimulating inflammatory responses and metabolic disorders in subchondral bone cells, further exacerbating bone changes and sclerosis [37]. Nevertheless, despite the close relationship of these tissues in the pathogenesis of OA, the distinctions among these tissues should also be acknowledged. In this study, the training dataset primarily consisted of subchondral bone tissues, whereas the validation process focused on cartilage and synovial tissues. This selection leads to the potential divergence of the analysis results, a factor that should be underscored to ensure readers’ awareness.

The roles SLC2A1 played in OA have been widely reported [24, 25, 38]. Guan et al. disclosed that the knockout of SLC2A1 promoted the levels of HIF-1alpha and apoptosis in chondrocytes [24]. Yao et al. and Zheng et al. reported that SLC2A1 could serve as the diagnosis biomarker in OA [25, 38], which was in accordance with our findings. The current study has identified a positive correlation between SLC2A1 and E2F-related signaling pathways. The E2F gene family plays a pivotal role in the regulation of the cell cycle and cell proliferation. E2F proteins function as transcription factors that govern the expression of genes involved in cell division, DNA replication, and cell differentiation [39]. These proteins are responsible for orchestrating the progression of cells through different phases of the cell cycle, ensuring appropriate cell growth and development. Dysregulation of E2F genes has been linked to various diseases, including osteoarthritis (OA), where abnormal E2F activity can result in uncontrolled cell apoptosis [40]. However, further investigation is required to elucidate the regulatory relationship between SLC2A1 and E2F-related signaling in the pathogenesis of OA.

NDUFB9 encodes a protein serving as a subunit of the mitochondrial oxidative phosphorylation complex I [41]. Therefore, the dysfunction of NDUFB9 leads to the overproduction of mitochondria-derived reactive oxygen species (mtROS) and the disturbance of the NAD+/NADH balance in tumor cells [42]. Nevertheless, no investigation of the biological functions of NDUFB9 in OA has been conducted so far. In this study, we reported that NDUFB9 was a significant diagnosis biomarker and could promote vitality and inhibit the apoptosis of chondrocytes in OA for the first time. Besides, the local samples indicated that NDUFB9 was significantly down-regulated in the cartilage tissues isolated from OA patients. The function enrichment analysis revealed a positive correlation between NDUFB9 and the activation of oxidative phosphorylation. Oxidative phosphorylation is a vital metabolic process in cells, primarily responsible for converting the chemical energy of organic molecules into a usable form of cellular energy, specifically adenosine triphosphate (ATP). Oxidative phosphorylation is the predominant pathway through which cells generate ATP and plays a critical role in maintaining cellular survival and function. A previous study reported an upregulation of oxidative phosphorylation activation levels in osteoarthritis (OA) samples, and inhibiting this process significantly impeded OA progression [43]. Our findings suggest a strong association between NDUFB9 and the dysregulation of oxidative phosphorylation in OA, although the underlying mechanisms remain unclear.

The current study developed the diagnostic models based on four common algorithms, namely LR, RF, SVM, and XGB. These algorithms each have their own characteristics [44]. The advantage of the LR model lies in its simplicity and speed, making it suitable for binary classification problems and performing well on linearly separable datasets. It also has strong interpretability. The RF model is effective in handling high-dimensional data and a large number of features, and it has good robustness. The SVM model can handle high-dimensional data and non-linear relationships, and it has good generalization ability. Especially in dealing with small samples and high-dimensional features, the SVM model performs well and can be used for non-linear mapping through kernel functions. The XGB model can handle various types of data and has good accuracy and generalization ability. It can handle high-dimensional data and non-linear relationships, has good robustness and interpretability, and can automatically handle missing and outlier values. In this study, we observed remarkable performance of the LR model in the validation-cartilage cohort (AUC = 0.726), whereas the RF model exhibited strong performance in both the validation-synovial (AUC = 0.736) and validation-local (AUC = 0.715) cohorts. These findings indicate the versatility of these models across various tissue types.

The limitations of this study should be acknowledged. First, despite the fact that the diagnosis values of SLC2A1 and NDUFB9 have been validated in multiple public cohorts and local clinical samples, a large-scale, multi-center, and prospective clinical trial would be more beneficial to clarify these genes’ usefulness. Second, although we have investigated the functions of NDUFB9 *in vitro*, the *in vivo* experiments and the exploration of its specific regulatory mechanisms remain needed.

## CONCLUSIONS

Collectively, a lactate metabolism-related gene signature was developed to diagnose OA, which was validated in multiple independent cohorts, local clinical samples, and cellular functional experiments. Our findings provide novel insights into the biological mechanisms of OA and offer a possible tool in clinical practice.

## Supplementary Material

Supplementary Figures

Supplementary Tables 1-5

Supplementary Table 6

Supplementary Tables 7-11

Supplementary Table 12
