# Supplementary material for: A lactate metabolism-related gene signature to diagnose osteoarthritis based on machine learning combined with experimental validation
Source: Aging (Albany NY). 2024 Oct 16;16(20):13076–103. doi: 10.18632/aging.205873 (PMC11552637; doi:10.18632/aging.205873)
Supplement: Supplementary Figures [file aging-16-205873-s001.pdf]

SUPPLEMENTARY FIGURES

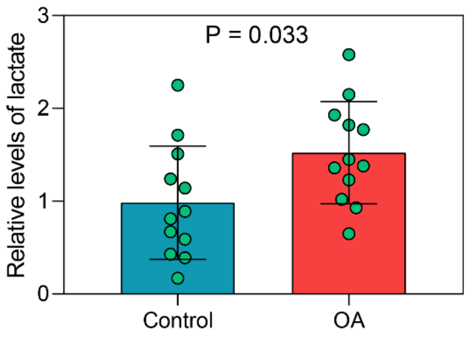

Supplementary Figure 1. The levels of lactate in the knee cartilage tissues collected from the control and OA subjects. Abbreviation: OA, osteoarthritis.

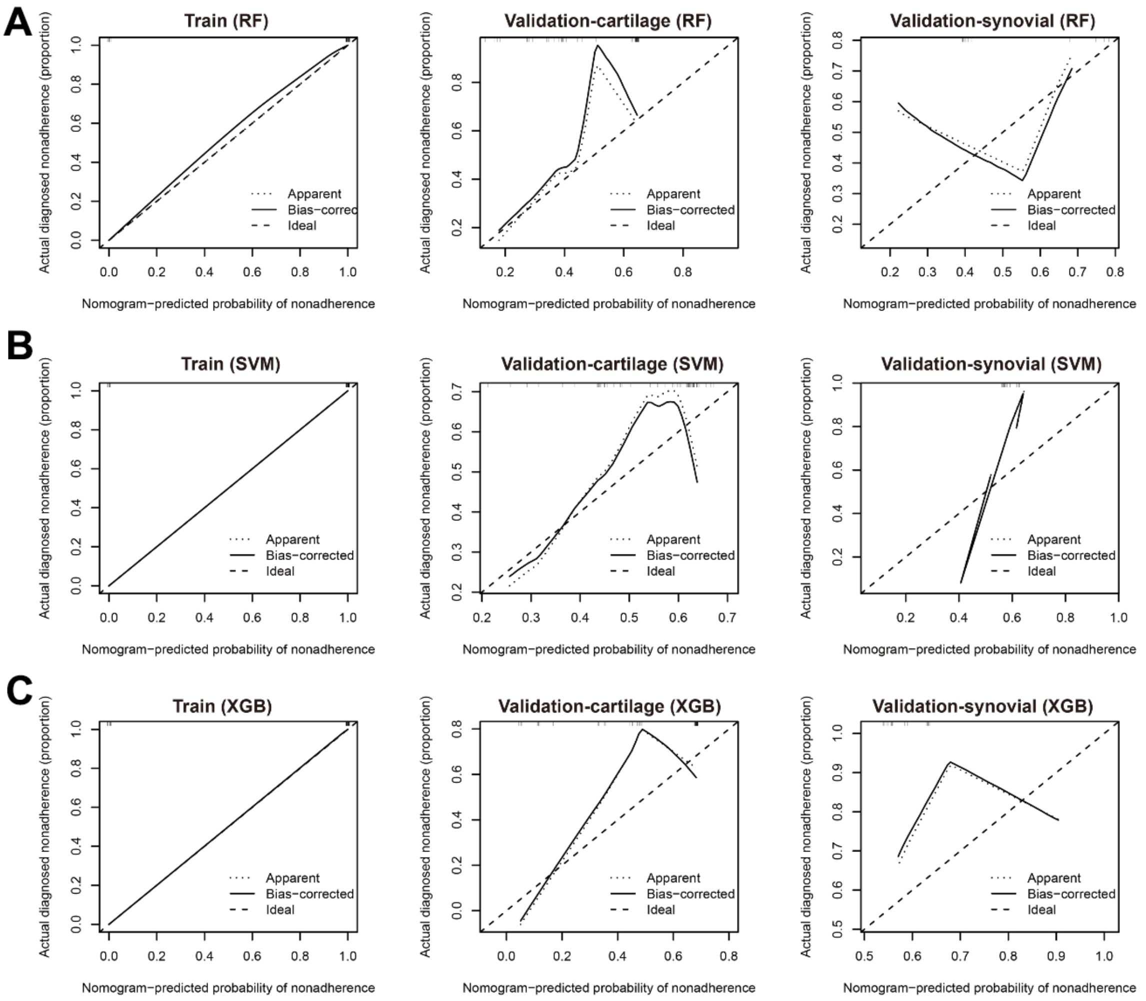

Supplementary Figure 2. The calibration analysis of the RF (**A**), SVM (**B**), and XGB (**C**) models. Abbreviations: RF, random forest; SVM, supporter vector machine; XGB, XGBoost.

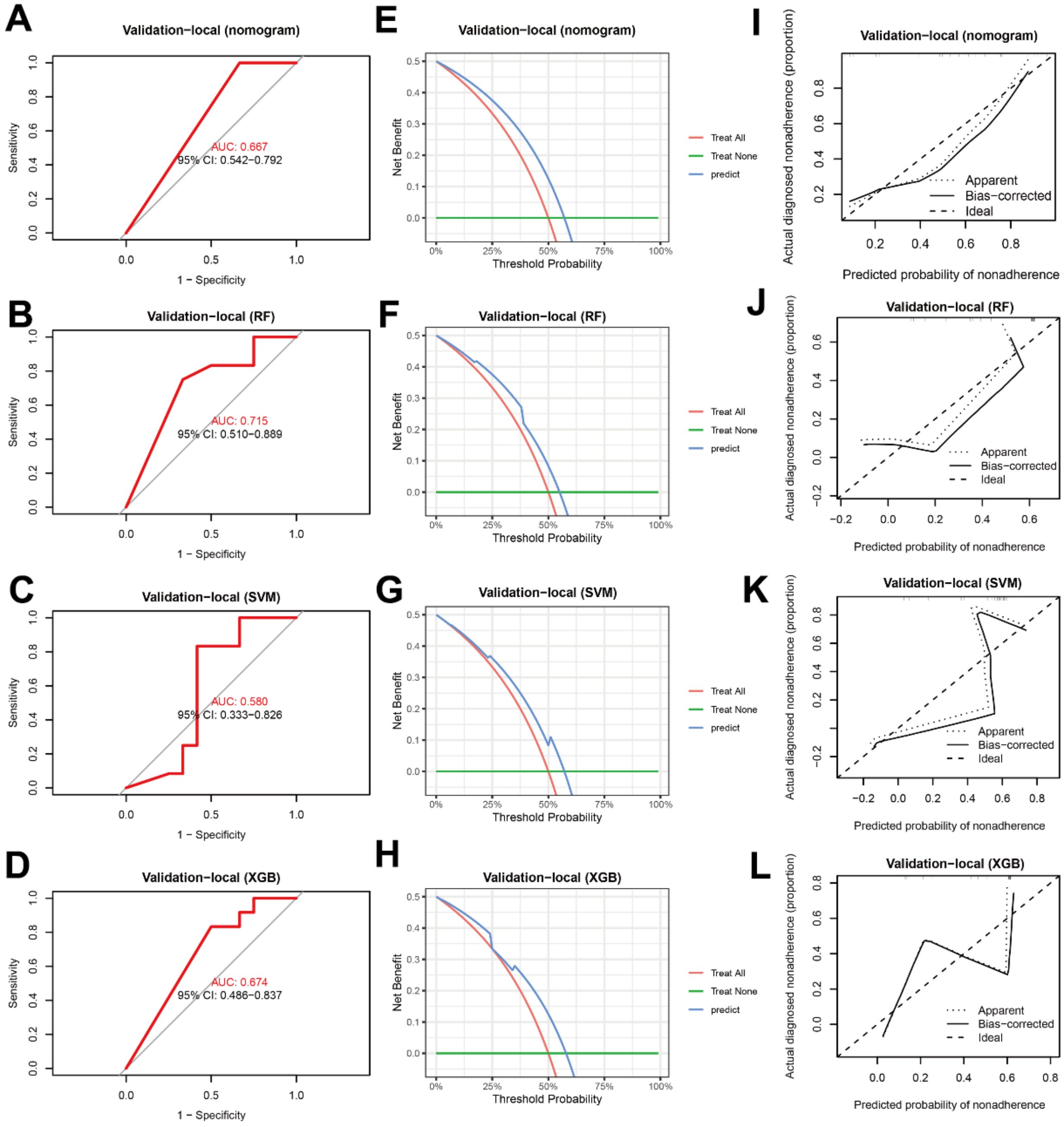

Supplementary Figure 3. The predictive performance of the established models in the local cohort. (**A**–**D**) The ROC analysis indicates the predictive performance of the nomogram (**A**), RF (**B**), SVM (**C**), and XGB (**D**) models in the validation-local cohort. (**E**–**H**) DCA displays the net benefit under different thresholds of the nomogram (**E**), RF (**F**), SVM (**G**), and XGB (**H**) models in the local cohort. (**I**–**L**) The calibration analysis shows the predictive performance of the nomogram (**I**), RF (**J**), SVM (**K**), and XGB (**L**) models in the local cohort. Abbreviations: ROC, receiver operating characteristic; DCA, decision curve analysis; AUC, area under curve; CI, confidence interval.

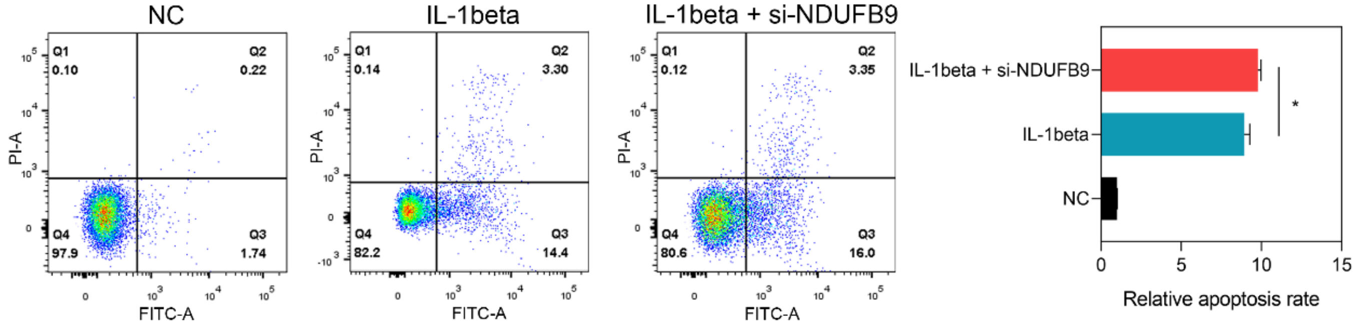

Supplementary Figure 4. Flow cytometry apoptosis analysis.

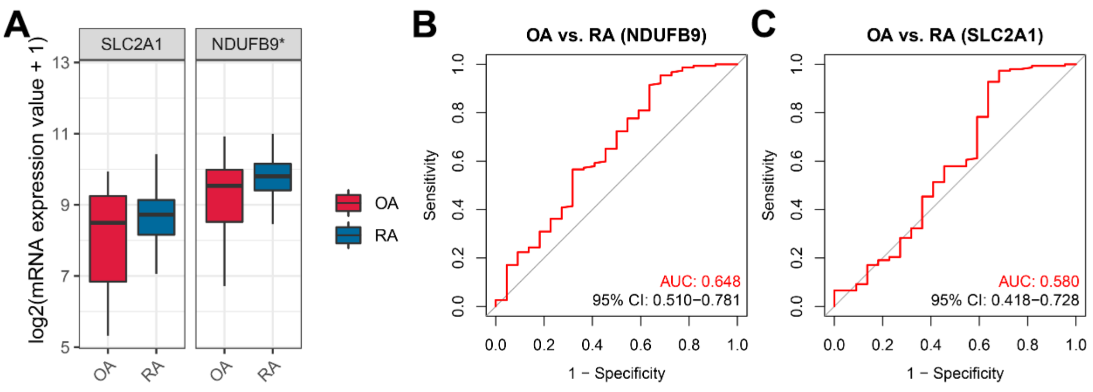

Supplementary Figure 5. The ability of SLC2A1 and NDUFB9 to distinguish OA from RA. (**A**) The expression levels of SLC2A1 and NDUFB9 in the joint synovial tissues from 22 OA and 152 RA samples. (**B**, **C**) ROC analysis indicates the ability of NDUFB9 (**B**) and SLC2A1 (**C**) to distinguish OA from RA.
