# Supplementary material for: A lactate metabolism-related gene signature to diagnose osteoarthritis based on machine learning combined with experimental validation
Source: Aging (Albany NY). 2024 Oct 16;16(20):13076–103. doi: 10.18632/aging.205873 (PMC11552637; doi:10.18632/aging.205873)
Supplement: Supplementary Tables 1-5 [file aging-16-205873-s002.pdf]

SUPPLEMENTARY TABLES

Supplementary Table 1. 273 lactate metabolism-related genes collected from the MSigDB.

Gene symbol
HMOX1, NGLY1, CLPB, NDUFB9, FKTN, MLIP, HIBCH, PDP1, SLC2A1, POMT1, SLC4A1, CYC1, COQ2, SLC16A8, CFI, SLC25A19, LDHB, RHAG, ALDOA, C1QBP, GATA1, SLC25A12, MRPL12, XK, NDUFB8, LDHA, NDUFAF3, TIMM50, NDUFV2, TUFM, EMB, COX8A, MYC, PYGL, MDH2, HTRA2, CYP27A1, HAGH, PLEC, GATA2, HSD17B10, PDSS1, SCO1, NDUFS2, CHCHD10, MTHFD1, MRPS22, GYS2, UQCRH, KLF1, SDHB, DGUOK, ISCA1, NDUFS1, AIFM1, RPS14, NDUFS6, HBB, PUS1, RNASEH1, COX5A, SUCLG1, JAK2, NDUFA6, ADAMTS13, NDUFA2, PIGA, PDHX, STAT2, SLC19A1, RHD, PNPLA8, IRAK1, MRPS7, UQCRC2, HPDL, ABCG8, COX6B1, COX4I1, POMT2, SOD1, NDUFAF2, NDUFA13, SFXN4, LIAS, LETM1, ZNFX1, LONP1, SLC25A10, SLC25A3, NDUFAF4, MRPS34, ACAT2, MRPS2, SLC5A8, COX10, ECHS1, PMPCB, PNPLA2, SLC16A1, ACAT1, PNPT1, NDUFB11, ATAD3A, RHCE, COQ4, NUBPL, PER2, FLI1, MRPL3, TRMT5, LRPPRC, SPP1, CAV3, RRM2B, SDHA, COX15, POMGNT1, DNM1L, NDUFS3, EARS2, MRPS28, LYST, INPP5K, WARS2, TK2, PNPO, NARS2, FASTKD2, YARS2, NDUFS8, SLC25A4, KY, GAA, NDUFA12, NDUFA1, MIPEP, GOT2, CA5A, ACAD9, ALDH4A1, NDUFB3, MPV17, PITRM1, NDUFS7, SLC16A7, AGK, UQCRQ, AARS2, PDHA1, TET2, DLD, SLC13A3, CDAN1, MVK, PHKG2, GTPBP3, PARK7, BCS1L, PLA2G6, LIPT2, FBXL4, OGDH, LYRM4, LIPT1, TRMU, PIK3CG, KIF23, NDUFA8, TXN2, SLC7A7, TSFM, POLRMT, NDUFB7, NFS1, KCNN4, VARS2, CALR, SLC16A3, COX6A2, FARS2, TCIRG1, NDUFA11, SIL1, NDUFB10, LYRM7, SLC19A3, OCRL, STAT4, CD46, LIPA, ATPAF2, DARS2, MPL, POLG2, TTC26, NDUFAF1, NSUN2, GFM2, PDSS2, GFM1, UQCRB, LPIN1, SURF1, COX16, MTO1, SYNJ1, PPCS, HLA-DRB1, SLC25A42, ACTN3, AKR1D1, CHEK2, VPS13A, PC, COL4A1, NDUFC2, MRPS14, SLC25A26, B3GALNT2, NDUFA4, HIF1A, TP53, DTYMK, CPT2, FKRP, RARS2, LDHC, FOXRED1, DAG1, RMND1, HMGCL, TMEM126B, COQ9, PIEZO1, TACO1, MECR, TIMMDC1, DNAJC19, NDUFA10, ISCU, SLC39A8, MECP2, TIMM22, HS6ST2, NSUN3, TPK1, HMGCS2, NDUFS4, CFH, ACADM, MTFMT, MRPS16, ALDH6A1, LDHAL6B, LDHD, SCO2, COG8, NFU1, SLC25A13, CARS2, TARS2, PNKD, NDUFV1, UPB1, LDHAL6A, SERAC1, RB1, POLG, NDUFA9, MRPL44, PFKFB2, SLC5A12.

Supplementary Table 2. The information of the datasets from the GEO.

ID	Dataset type	Tissues	Platform	Experimental type	Sample size (Control/OA)	PMID
GSE51588	Training	Cartilage tissue	GPL13497	Expression profiling by array	10/40	24229462
GSE114007	Validation	Cartilage tissue	GPL11154<br>GPL18573	Expression profiling by high throughput sequencing	18/20	30081074
GSE117999	Validation	Cartilage tissue	GPL20844	Expression profiling by array	12/12	NA
GSE82107	Validation	Synovial tissue	GPL570	Expression profiling by array	7/10	27870898
GSE89408	-	Synovial tissue	GPL11154	Expression profiling by high throughput sequencing	OA: 22<br>RA: 152	28455435<br>28863153

Supplementary Table 3. The baseline clinicopathological features of the control and OA subjects in the local cohort.

Feature	Control (N = 12)	OA (N = 12)
Age	54.17 ± 7.8	60.08 ± 8.1
Sex		
Male	8 (66.67%)	6 (50.00%)
Female	4 (33.33%)	6 (50.00%)
BMI	29.17 ± 7.5	32.08 ± 9.4
Kellgren-Lawrence Grade		
I	-	0 (0.00%)
II	-	0 (0.00%)
III	-	3 (25.00%)
IV	-	9 (75.00%)

Supplementary Table 4. The siRNA sequences used in this study.

ID	Sequence (5'-3')
si-NDUFB9-1	GTCCCAGAATGGTGCTTAGAT
si-NC-1	GCGTGTCCAATTCGAGTAGAT
si-NDUFB9-2	GGCAATGTATCCTGATTACTT
si-NC-2	GACTTACTCGGCGTTATAATT

Supplementary Table 5. The primer sequence of the qPCR experiments.

Gene	Sequence (5'-3')
SLC2A1	Forward Primer: GGCCAAGAGTGTGCTAAAGAA
	Reverse Primer: ACAGCGTTGATGCCAGACAG
NDUFB9	Forward Primer: GTGGTGCGTCCAGAGAGAC
	Reverse Primer: GGCCTTCGCCATATCCTTTTC
GAPDH	Forward Primer: GGAGCGAGATCCCTCCAAAAT
	Reverse Primer: GGCTGTTGTCATACTTCTCATGG
