# Supplementary material for: A lactate metabolism-related gene signature to diagnose osteoarthritis based on machine learning combined with experimental validation
Source: Aging (Albany NY). 2024 Oct 16;16(20):13076–103. doi: 10.18632/aging.205873 (PMC11552637; doi:10.18632/aging.205873)
Supplement: Supplementary Table 6 [file aging-16-205873-s003.docx]

Supplementary Table 6. The expression difference of 273 lactate metabolism-related genes between the control and OA samples.

| **Gene** | **logFC** | **AveExpr** | **t** | **P value** | **FDR** | **B** |
| --- | --- | --- | --- | --- | --- | --- |
| HMOX1 | -1.569332882 | -0.149583759 | -8.096144057 | 9.36E-11 | 2.56E-08 | 14.3441232 |
| NGLY1 | -0.475650289 | 0.023893186 | -7.741522931 | 3.39E-10 | 4.63E-08 | 13.07896114 |
| CLPB | -0.967339354 | 0.144124155 | -7.485678956 | 8.62E-10 | 7.85E-08 | 12.16258679 |
| NDUFB9 | -0.846779906 | 0.036398629 | -7.354276989 | 1.39E-09 | 9.51E-08 | 11.69112017 |
| FKTN | 0.819500753 | 0.037822899 | 7.225914863 | 2.23E-09 | 1.22E-07 | 11.23020792 |
| MLIP | 1.177548992 | -0.025502681 | 7.104952227 | 3.47E-09 | 1.58E-07 | 10.79567612 |
| HIBCH | 0.679622221 | -0.020322332 | 6.883198969 | 7.82E-09 | 3.05E-07 | 9.999030797 |
| PDP1 | 0.970083582 | -0.131201766 | 6.791860877 | 1.09E-08 | 3.45E-07 | 9.671041355 |
| SLC2A1 | -1.120930454 | 0.040176946 | -6.781015403 | 1.14E-08 | 3.45E-07 | 9.632106194 |
| POMT1 | 0.658443563 | 0.005010415 | 6.750006969 | 1.27E-08 | 3.47E-07 | 9.520800589 |
| SLC4A1 | -2.264151183 | 0.189519787 | -6.051762567 | 1.63E-07 | 3.61E-06 | 7.025738001 |
| CYC1 | -0.929025742 | -0.01072577 | -6.036625959 | 1.72E-07 | 3.61E-06 | 6.972018954 |
| COQ2 | -0.677101996 | 0.018099003 | -6.035586561 | 1.73E-07 | 3.61E-06 | 6.968330965 |
| SLC16A8 | 1.212001321 | -0.110889113 | 6.016334713 | 1.85E-07 | 3.61E-06 | 6.900040037 |
| CFI | 1.100331943 | 0.188028457 | 5.979812035 | 2.11E-07 | 3.84E-06 | 6.770583511 |
| SLC25A19 | -0.414218861 | 0.031803487 | -5.954532726 | 2.32E-07 | 3.95E-06 | 6.681057348 |
| LDHB | -0.856377163 | 0.027914114 | -5.770224608 | 4.51E-07 | 7.24E-06 | 6.03043504 |
| RHAG | -3.933405772 | 0.677685599 | -5.67159673 | 6.43E-07 | 9.75E-06 | 5.683957349 |
| ALDOA | -0.697491912 | -0.010667915 | -5.624342909 | 7.62E-07 | 1.09E-05 | 5.518418251 |
| C1QBP | -0.902647124 | -0.036151673 | -5.539843942 | 1.03E-06 | 1.41E-05 | 5.223203013 |
| GATA1 | -2.710516868 | 0.451733768 | -5.525848148 | 1.08E-06 | 1.41E-05 | 5.174409126 |
| SLC25A12 | 0.533157157 | -0.050551091 | 5.480559898 | 1.27E-06 | 1.53E-05 | 5.016729342 |
| MRPL12 | -0.766617633 | 0.05584466 | -5.477437593 | 1.29E-06 | 1.53E-05 | 5.005870449 |
| XK | -2.743504324 | 0.43251065 | -5.423290044 | 1.56E-06 | 1.78E-05 | 4.817807219 |
| NDUFB8 | -0.456293882 | -0.018485355 | -5.38763481 | 1.78E-06 | 1.94E-05 | 4.694239216 |
| LDHA | -1.463292818 | 0.167878878 | -5.364557877 | 1.93E-06 | 2.02E-05 | 4.614380065 |
| NDUFAF3 | -0.561439034 | -0.01490574 | -5.341879683 | 2.09E-06 | 2.11E-05 | 4.535992316 |
| TIMM50 | -0.533622647 | 0.060515005 | -5.272620278 | 2.67E-06 | 2.54E-05 | 4.297174007 |
| NDUFV2 | -0.652330406 | -0.026174855 | -5.269689576 | 2.70E-06 | 2.54E-05 | 4.28708813 |
| TUFM | -0.546588994 | -0.008634644 | -5.208399151 | 3.35E-06 | 3.00E-05 | 4.076538611 |
| EMB | -1.351308411 | 0.23040942 | -5.203938906 | 3.41E-06 | 3.00E-05 | 4.061245126 |
| COX8A | -0.683914831 | -0.031380404 | -5.090916252 | 5.07E-06 | 4.32E-05 | 3.675063869 |
| MYC | -0.858837953 | -0.055718841 | -5.065261019 | 5.55E-06 | 4.59E-05 | 3.587779513 |
| PYGL | -1.059790447 | -0.254083789 | -5.045105053 | 5.95E-06 | 4.78E-05 | 3.5193059 |
| MDH2 | -0.583669304 | -0.048177146 | -5.018352108 | 6.53E-06 | 5.10E-05 | 3.428560807 |
| HTRA2 | -0.625718347 | -0.013298894 | -4.958680279 | 8.05E-06 | 6.10E-05 | 3.22674385 |
| CYP27A1 | 0.604427165 | -0.088312565 | 4.940919361 | 8.56E-06 | 6.32E-05 | 3.166834807 |
| HAGH | -0.898048948 | 0.201507892 | -4.881413965 | 1.05E-05 | 7.56E-05 | 2.966670627 |
| PLEC | 0.737279775 | 0.056138855 | 4.82693489 | 1.27E-05 | 8.90E-05 | 2.784181358 |
| GATA2 | 1.069267832 | 0.048691959 | 4.771369186 | 1.54E-05 | 0.000105081 | 2.598835087 |
| HSD17B10 | -0.663937137 | -0.013604296 | -4.748921562 | 1.66E-05 | 0.000110749 | 2.524188655 |
| PDSS1 | -0.481039731 | 0.022290372 | -4.730538752 | 1.77E-05 | 0.000115161 | 2.463159883 |
| SCO1 | -0.449905069 | 0.045304747 | -4.722925432 | 1.82E-05 | 0.000115461 | 2.437911361 |
| NDUFS2 | -0.80567594 | -0.001895865 | -4.700505558 | 1.96E-05 | 0.000120642 | 2.363651056 |
| CHCHD10 | -0.600504489 | 0.064381428 | -4.696863949 | 1.99E-05 | 0.000120642 | 2.351602192 |
| MTHFD1 | -0.531830953 | 0.065139638 | -4.618814929 | 2.60E-05 | 0.000154079 | 2.094260231 |
| MRPS22 | -0.385242583 | -0.005951023 | -4.603081194 | 2.74E-05 | 0.000159099 | 2.042594571 |
| GYS2 | -1.50641343 | 0.152635607 | -4.595389976 | 2.81E-05 | 0.000159913 | 2.017364843 |
| UQCRH | -0.68075764 | -0.02820716 | -4.541413241 | 3.38E-05 | 0.00018814 | 1.840796782 |
| KLF1 | -1.994048146 | 0.340095626 | -4.533152279 | 3.47E-05 | 0.000189606 | 1.813850842 |
| SDHB | -0.755278293 | -0.091449127 | -4.526767843 | 3.55E-05 | 0.000189716 | 1.793040056 |
| DGUOK | -0.420781647 | -0.007631428 | -4.521386247 | 3.61E-05 | 0.000189716 | 1.775507811 |
| ISCA1 | -0.514540548 | -0.041567799 | -4.490380324 | 4.01E-05 | 0.000206684 | 1.674669906 |
| NDUFS1 | -0.364351652 | 0.008362655 | -4.473620888 | 4.25E-05 | 0.000214646 | 1.620289067 |
| AIFM1 | -0.417051959 | 0.034750328 | -4.457931943 | 4.48E-05 | 0.000222172 | 1.569461807 |
| RPS14 | -0.480087857 | 0.0372159 | -4.438583641 | 4.78E-05 | 0.000232868 | 1.506887006 |
| NDUFS6 | -0.463975119 | -0.001757851 | -4.383646641 | 5.74E-05 | 0.000275026 | 1.329872511 |
| HBB | -1.40743368 | -0.237647881 | -4.375718293 | 5.90E-05 | 0.000277541 | 1.304407921 |
| PUS1 | -0.613525539 | 0.042071133 | -4.334589266 | 6.76E-05 | 0.000312949 | 1.172644612 |
| RNASEH1 | -0.56529146 | -0.055100677 | -4.313635823 | 7.25E-05 | 0.000329943 | 1.105736656 |
| COX5A | -0.636415217 | -0.031387979 | -4.300647012 | 7.57E-05 | 0.000338838 | 1.064336577 |
| SUCLG1 | -0.505807467 | 0.036634105 | -4.159034822 | 0.000120764 | 0.000531391 | 0.616828249 |
| JAK2 | -0.625572389 | 0.045789004 | -4.154354658 | 0.000122629 | 0.000531391 | 0.602162527 |
| NDUFA6 | -0.669255186 | -0.021757353 | -4.146015252 | 0.000126021 | 0.000537558 | 0.57605042 |
| ADAMTS13 | 0.608019186 | -0.022492518 | 4.125796853 | 0.000134627 | 0.000565433 | 0.512851081 |
| NDUFA2 | -0.461324048 | -0.001106321 | -4.112216474 | 0.000140725 | 0.000580969 | 0.470487472 |
| PIGA | -0.512383893 | 0.060505322 | -4.108192308 | 0.000142582 | 0.000580969 | 0.457947618 |
| PDHX | -0.533328039 | 0.052303982 | -4.006344688 | 0.000198348 | 0.000796309 | 0.142658445 |
| STAT2 | 0.499762249 | 0.053658065 | 3.954073879 | 0.000234641 | 0.00092836 | -0.017560715 |
| SLC19A1 | -0.459326635 | -0.05385252 | -3.938848799 | 0.000246366 | 0.000960826 | -0.064019262 |
| RHD | -1.89919645 | 0.271941698 | -3.931553171 | 0.000252182 | 0.000969659 | -0.086247693 |
| PNPLA8 | -0.540447023 | 0.026221446 | -3.907928456 | 0.000271942 | 0.001031114 | -0.158076473 |
| IRAK1 | -0.438788911 | 0.036529379 | -3.882466292 | 0.000294909 | 0.001102878 | -0.235230628 |
| MRPS7 | -0.530704712 | -0.057853203 | -3.818728717 | 0.000360889 | 0.001331387 | -0.427156163 |
| UQCRC2 | -0.33861928 | 0.007750664 | -3.804255144 | 0.00037774 | 0.001374973 | -0.470494284 |
| HPDL | -0.523064754 | -0.080457134 | -3.795496783 | 0.000388302 | 0.00139482 | -0.49667487 |
| ABCG8 | 0.738361746 | 0.035569838 | 3.763359834 | 0.000429542 | 0.00152292 | -0.5924491 |
| COX6B1 | -0.463872983 | -0.066539725 | -3.734529859 | 0.000470092 | 0.001645322 | -0.677976241 |
| COX4I1 | -0.510230586 | -0.067294442 | -3.712251901 | 0.000503918 | 0.001741387 | -0.743809321 |
| POMT2 | 0.391519316 | -0.007841865 | 3.679090694 | 0.000558625 | 0.001888111 | -0.84138349 |
| SOD1 | -0.392107898 | 0.036835824 | -3.67817794 | 0.000560209 | 0.001888111 | -0.844062038 |
| NDUFAF2 | -0.578711981 | 0.106961535 | -3.669233779 | 0.000575955 | 0.001917509 | -0.870288941 |
| NDUFA13 | -0.391918753 | -0.007186491 | -3.650783019 | 0.000609788 | 0.002005688 | -0.924274459 |
| SFXN4 | -0.260565806 | -0.02306427 | -3.638677458 | 0.000633011 | 0.002057284 | -0.959607912 |
| LIAS | 0.317471789 | -0.035741768 | 3.6190741 | 0.000672422 | 0.00215966 | -1.016679401 |
| LETM1 | -0.48677016 | 0.092877189 | -3.605439434 | 0.000701206 | 0.00222592 | -1.056266764 |
| ZNFX1 | 0.42935136 | -0.022868225 | 3.595773304 | 0.000722321 | 0.002266595 | -1.084278045 |
| LONP1 | -0.555885526 | -0.017451161 | -3.540123335 | 0.000856215 | 0.002656212 | -1.244668816 |
| SLC25A10 | -0.41954119 | -0.029579602 | -3.522118936 | 0.000904387 | 0.002774131 | -1.29623661 |
| SLC25A3 | -0.338396955 | 0.021559867 | -3.518095008 | 0.000915501 | 0.002777021 | -1.30774001 |
| NDUFAF4 | -0.545692286 | 0.092262675 | -3.421900207 | 0.001223383 | 0.003660825 | -1.580322969 |
| MRPS34 | -0.362366874 | 0.019902077 | -3.419097548 | 0.001233685 | 0.003660825 | -1.588194308 |
| ACAT2 | -0.24926343 | -0.006045169 | -3.347122538 | 0.001528277 | 0.004473454 | -1.78894054 |
| MRPS2 | -0.239258457 | 0.029922471 | -3.344471407 | 0.00154031 | 0.004473454 | -1.796282951 |
| SLC5A8 | -0.611526157 | 0.080667704 | -3.271471684 | 0.001909146 | 0.005486281 | -1.996982502 |
| COX10 | -0.524261592 | -0.104520275 | -3.23351076 | 0.002132514 | 0.006064335 | -2.100207081 |
| ECHS1 | -0.384925759 | 0.019077465 | -3.209430004 | 0.002286756 | 0.006435922 | -2.165276266 |
| PMPCB | 0.404814886 | -0.006098662 | 3.205756737 | 0.002311189 | 0.006438312 | -2.175173547 |
| PNPLA2 | -0.862846184 | -0.117680394 | -3.197230349 | 0.002368854 | 0.0064849 | -2.198118065 |
| SLC16A1 | -0.526874895 | 0.035109758 | -3.196271737 | 0.002375421 | 0.0064849 | -2.200695149 |
| ACAT1 | -0.358460081 | 0.010709307 | -3.178288866 | 0.002501843 | 0.006709335 | -2.248943774 |
| PNPT1 | -0.309065991 | 0.02362073 | -3.177603526 | 0.002506785 | 0.006709335 | -2.250778965 |
| NDUFB11 | -0.425861576 | -0.069347267 | -3.160408987 | 0.002633801 | 0.00694463 | -2.296735304 |
| ATAD3A | -0.477243632 | 0.081973571 | -3.158855025 | 0.002645573 | 0.00694463 | -2.300880372 |
| RHCE | -2.225266101 | 0.587710459 | -3.130207719 | 0.002871668 | 0.007466336 | -2.377048298 |
| COQ4 | 0.418801469 | -0.081917347 | 3.114715865 | 0.003001376 | 0.007729958 | -2.418042448 |
| NUBPL | 0.399686076 | 0.092448406 | 3.107886197 | 0.003060288 | 0.007808024 | -2.43607101 |
| PER2 | 0.580860913 | -0.08632479 | 3.103090563 | 0.003102301 | 0.007841927 | -2.448714131 |
| FLI1 | -0.361883476 | 0.051057662 | -3.098571241 | 0.003142388 | 0.007870385 | -2.460616597 |
| MRPL3 | -0.550203328 | 0.052284527 | -3.089339427 | 0.003225791 | 0.008005826 | -2.484893422 |
| TRMT5 | -0.293825046 | 0.039104691 | -3.058420505 | 0.003520481 | 0.008658481 | -2.565838385 |
| LRPPRC | -0.269327604 | -0.015067993 | -3.053018538 | 0.003574486 | 0.008712811 | -2.579923034 |
| SPP1 | 0.73560945 | -0.03351551 | 3.029096187 | 0.003823098 | 0.009236335 | -2.642088964 |
| CAV3 | 0.516934438 | 0.050218903 | 3.016158441 | 0.0039642 | 0.009493216 | -2.675568103 |
| RRM2B | 0.337096663 | 0.006302864 | 3.010709307 | 0.004025074 | 0.009555175 | -2.689638991 |
| SDHA | -0.285927982 | -0.042244719 | -2.946175768 | 0.004815542 | 0.011333129 | -2.854918659 |
| COX15 | 0.268042295 | 0.019320919 | 2.895699849 | 0.005532126 | 0.012908294 | -2.982421998 |
| POMGNT1 | 0.214970864 | -0.002993889 | 2.883801167 | 0.005714896 | 0.013221751 | -3.012248462 |
| DNM1L | -0.224524486 | -0.040711044 | -2.834962625 | 0.006525334 | 0.014969884 | -3.133742057 |
| NDUFS3 | -0.270533013 | -0.035531177 | -2.825019639 | 0.006702852 | 0.015248987 | -3.158292118 |
| EARS2 | 0.419003774 | 0.001588431 | 2.800753562 | 0.007155005 | 0.016143109 | -3.217942405 |
| MRPS28 | -0.306951786 | 0.016313343 | -2.793607416 | 0.007293435 | 0.016320555 | -3.235436996 |
| LYST | 0.446979787 | -0.053254586 | 2.732912744 | 0.008572756 | 0.019027336 | -3.382692831 |
| INPP5K | -0.21291978 | 0.013122312 | -2.714997665 | 0.008988083 | 0.019788281 | -3.425698309 |
| WARS2 | -0.3408331 | 0.013164778 | -2.657859969 | 0.010439302 | 0.022784588 | -3.561438932 |
| TK2 | 0.49946947 | 0.085283813 | 2.653931556 | 0.010546566 | 0.022784588 | -3.570691515 |
| PNPO | -0.269707081 | 0.006324292 | -2.65200914 | 0.010599424 | 0.022784588 | -3.5752156 |
| NARS2 | -0.311223348 | -0.066127749 | -2.619556988 | 0.011529076 | 0.024589357 | -3.651210198 |
| FASTKD2 | -0.17773709 | -0.001744251 | -2.575447533 | 0.012912133 | 0.027325677 | -3.753355601 |
| YARS2 | -0.265368297 | 0.000598375 | -2.568911846 | 0.013129451 | 0.027571846 | -3.768377047 |
| NDUFS8 | -0.331295744 | 0.044675052 | -2.551991701 | 0.013707608 | 0.028566236 | -3.807128954 |
| SLC25A4 | -0.437042918 | -0.126621886 | -2.535110303 | 0.014307409 | 0.029590323 | -3.845594523 |
| KY | 0.454649949 | -0.143943809 | 2.5178381 | 0.014945661 | 0.030558029 | -3.884745274 |
| GAA | 0.332298947 | -0.01311802 | 2.516420378 | 0.014999179 | 0.030558029 | -3.88794956 |
| NDUFA12 | -0.304688379 | 0.025116119 | -2.494524714 | 0.01584808 | 0.032048339 | -3.937258408 |
| NDUFA1 | -0.30669221 | -0.003338599 | -2.443767668 | 0.017985464 | 0.036103174 | -4.050260873 |
| MIPEP | -0.217951577 | 0.012508192 | -2.417743936 | 0.019179205 | 0.038218416 | -4.107486499 |
| GOT2 | -0.222302388 | -0.025202046 | -2.398120603 | 0.020125984 | 0.039814447 | -4.150315692 |
| CA5A | 0.332310774 | -0.022840616 | 2.388519916 | 0.020604314 | 0.040467466 | -4.171168409 |
| ACAD9 | -0.217780203 | 0.009560751 | -2.345493409 | 0.022875622 | 0.044607462 | -4.263798291 |
| ALDH4A1 | -0.417075707 | 0.053394881 | -2.299059646 | 0.025575565 | 0.049351673 | -4.362238669 |
| NDUFB3 | -0.345364111 | 0.009526709 | -2.297514433 | 0.025670101 | 0.049351673 | -4.365487111 |
| MPV17 | 0.168434877 | 0.017502179 | 2.271419412 | 0.027314243 | 0.052145373 | -4.420077003 |
| PITRM1 | -0.220937643 | -0.042667656 | -2.263115045 | 0.02785674 | 0.052811736 | -4.437342711 |
| NDUFS7 | -0.416427328 | -0.061712548 | -2.24836213 | 0.028844113 | 0.054306503 | -4.467888022 |
| SLC16A7 | -0.45560285 | -0.008039922 | -2.242698606 | 0.029231319 | 0.054658561 | -4.479570618 |
| AGK | 0.21830268 | -0.038383284 | 2.238436565 | 0.029525735 | 0.054833508 | -4.488346318 |
| UQCRQ | -0.31438813 | -0.017716675 | -2.228242438 | 0.030240603 | 0.055781654 | -4.509280747 |
| AARS2 | 0.229995379 | 0.014072345 | 2.17906091 | 0.033908729 | 0.062128073 | -4.609170022 |
| PDHA1 | -0.257377617 | 0.009021913 | -2.1644228 | 0.03507359 | 0.063833935 | -4.638543556 |
| TET2 | -0.150578562 | 0.038709984 | -2.074012299 | 0.043076913 | 0.077880776 | -4.816290099 |
| DLD | -0.259347345 | -0.050290603 | -2.068662916 | 0.043596698 | 0.078301965 | -4.826606924 |
| SLC13A3 | 0.34981143 | -0.04276295 | 2.04768814 | 0.045687698 | 0.081521187 | -4.866841275 |
| CDAN1 | -0.222813664 | -0.032603644 | -2.024477798 | 0.048102396 | 0.085272429 | -4.910958139 |
| MVK | 0.146174026 | 0.019073351 | 1.95568436 | 0.055917771 | 0.098487428 | -5.039190135 |
| PHKG2 | -0.316993615 | -0.034727113 | -1.942658611 | 0.057514236 | 0.100649913 | -5.063041459 |
| GTPBP3 | 0.253924254 | 0.076599217 | 1.921178147 | 0.060232108 | 0.10413754 | -5.102073531 |
| PARK7 | -0.186632178 | 0.015085679 | -1.920485567 | 0.060321532 | 0.10413754 | -5.103325772 |
| BCS1L | 0.193063952 | 0.092366487 | 1.917937296 | 0.060651534 | 0.10413754 | -5.107929887 |
| PLA2G6 | 0.228668847 | -0.013263985 | 1.903338411 | 0.062571912 | 0.106763325 | -5.134204467 |
| LIPT2 | 0.243318451 | -0.019902888 | 1.895611414 | 0.063609125 | 0.107858951 | -5.148040762 |
| FBXL4 | 0.141259311 | -0.037191105 | 1.881059683 | 0.065602144 | 0.110017382 | -5.173964882 |
| OGDH | 0.189320887 | -0.007113486 | 1.880384198 | 0.065695932 | 0.110017382 | -5.175164048 |
| LYRM4 | -0.200902033 | 0.024543819 | -1.877547568 | 0.066091028 | 0.110017382 | -5.180195724 |
| LIPT1 | 0.282673846 | -0.030250112 | 1.866874182 | 0.067595754 | 0.111840248 | -5.199069088 |
| TRMU | -0.219413051 | 0.045033158 | -1.858249243 | 0.06883279 | 0.113200914 | -5.214251674 |
| PIK3CG | -0.373748604 | 0.061387291 | -1.848653515 | 0.070231506 | 0.114809587 | -5.231070965 |
| KIF23 | -0.676301254 | 0.040529242 | -1.807630468 | 0.07648466 | 0.124287572 | -5.302114725 |
| NDUFA8 | -0.211557073 | -0.009237137 | -1.801488509 | 0.077459956 | 0.125127622 | -5.312630784 |
| TXN2 | -0.173018073 | -0.071071415 | -1.795373532 | 0.078441305 | 0.125967508 | -5.323069327 |
| SLC7A7 | -0.218614507 | 0.014160405 | -1.764061897 | 0.083630818 | 0.132856895 | -5.376028576 |
| TSFM | -0.216630195 | 0.018307991 | -1.76362791 | 0.08370471 | 0.132856895 | -5.376756815 |
| POLRMT | -0.260421137 | 0.081857225 | -1.750089565 | 0.086037255 | 0.135769772 | -5.399394712 |
| NDUFB7 | -0.178704454 | 0.046178474 | -1.723947161 | 0.090694355 | 0.142201601 | -5.442669847 |
| NFS1 | 0.123508662 | 0.015560483 | 1.718959385 | 0.091606193 | 0.142201601 | -5.450860602 |
| KCNN4 | 0.63448067 | 0.113095361 | 1.718580552 | 0.091675757 | 0.142201601 | -5.451481844 |
| VARS2 | -0.15296042 | 0.003280639 | -1.70462659 | 0.094268713 | 0.145397507 | -5.474279688 |
| CALR | -0.245483348 | 0.027075138 | -1.691187389 | 0.096822925 | 0.148498081 | -5.496079656 |
| SLC16A3 | -0.558656512 | -0.108066807 | -1.679248035 | 0.099139609 | 0.15120175 | -5.515317249 |
| COX6A2 | 0.387790085 | 0.0597332 | 1.671604229 | 0.10064656 | 0.152647283 | -5.527569445 |
| FARS2 | -0.138226725 | -0.029834538 | -1.644650353 | 0.106110989 | 0.160045856 | -5.570373158 |
| TCIRG1 | 0.367977236 | 0.044038088 | 1.632043087 | 0.108748773 | 0.16312316 | -5.590179128 |
| NDUFA11 | -0.185883499 | -0.001211052 | -1.58293146 | 0.119537299 | 0.178326134 | -5.666020492 |
| SIL1 | 0.134559248 | 0.017325574 | 1.525758184 | 0.133166258 | 0.197578197 | -5.751659916 |
| NDUFB10 | -0.147190833 | -0.001341076 | -1.501546372 | 0.139299049 | 0.205560219 | -5.787060101 |
| LYRM7 | 0.345756777 | 0.017561149 | 1.486299926 | 0.143274392 | 0.210186864 | -5.80908593 |
| SLC19A3 | -0.663446461 | -0.218267075 | -1.482029303 | 0.144403826 | 0.210186864 | -5.815218546 |
| OCRL | -0.163961743 | 0.025875396 | -1.480747945 | 0.144744067 | 0.210186864 | -5.817055417 |
| STAT4 | 0.264615203 | -0.076986597 | 1.459711293 | 0.150420802 | 0.216005925 | -5.847003477 |
| CD46 | 0.307885657 | 0.093074237 | 1.457515117 | 0.151023377 | 0.216005925 | -5.850107267 |
| LIPA | -0.300614545 | -0.013192328 | -1.457145324 | 0.151125024 | 0.216005925 | -5.850629462 |
| ATPAF2 | -0.140043295 | -0.020388822 | -1.441574223 | 0.155454047 | 0.221036223 | -5.872507057 |
| DARS2 | -0.251042611 | -0.083139475 | -1.42932081 | 0.158928333 | 0.224805362 | -5.889570873 |
| MPL | 0.206511131 | -0.069862303 | 1.423705632 | 0.160540513 | 0.225915259 | -5.89734551 |
| POLG2 | -0.144075609 | -0.047109299 | -1.420789877 | 0.161382662 | 0.225935727 | -5.901371441 |
| TTC26 | 0.317113072 | 0.155743426 | 1.289961985 | 0.202805468 | 0.282479045 | -6.074108982 |
| NDUFAF1 | -0.133993219 | 0.04885086 | -1.255171884 | 0.215061589 | 0.298029511 | -6.117418316 |
| NSUN2 | -0.135582334 | -0.033696746 | -1.243825423 | 0.219175344 | 0.302196307 | -6.131302572 |
| GFM2 | 0.120688412 | 0.037593227 | 1.211221374 | 0.231319295 | 0.316040689 | -6.170537658 |
| PDSS2 | 0.104880667 | -0.040120906 | 1.207190447 | 0.23285422 | 0.316040689 | -6.17532009 |
| GFM1 | -0.090418628 | -0.022765603 | -1.204246469 | 0.233979938 | 0.316040689 | -6.178803413 |
| UQCRB | -0.128729551 | 0.01263218 | -1.201989483 | 0.234845649 | 0.316040689 | -6.181468442 |
| LPIN1 | -0.145108005 | 0.003824913 | -1.201575704 | 0.235004615 | 0.316040689 | -6.181956517 |
| SURF1 | -0.090676046 | 0.045351428 | -1.195831404 | 0.237219567 | 0.317455597 | -6.18871581 |
| COX16 | -0.13757782 | -0.007352008 | -1.145648349 | 0.257217317 | 0.342538183 | -6.246460558 |
| MTO1 | -0.173395047 | 0.042285243 | -1.133639873 | 0.26217634 | 0.347447286 | -6.259930052 |
| SYNJ1 | -0.166885995 | -0.08841092 | -1.113982256 | 0.270440166 | 0.356667465 | -6.281687837 |
| PPCS | -0.110079756 | 0.020643674 | -1.103682605 | 0.274842665 | 0.360730997 | -6.292943126 |
| HLA-DRB1 | 0.182431866 | -0.021470031 | 1.090661303 | 0.280480263 | 0.366368957 | -6.307029867 |
| SLC25A42 | -0.140297101 | -0.110893144 | -1.052265217 | 0.297572435 | 0.386844166 | -6.347637314 |
| ACTN3 | -0.180429958 | -0.048368952 | -1.038263297 | 0.303980223 | 0.393301425 | -6.362098983 |
| AKR1D1 | -0.263971369 | 0.076229302 | -1.029703561 | 0.307943606 | 0.39655002 | -6.370848296 |
| CHEK2 | -0.187245756 | -0.034516811 | -1.005065748 | 0.319547269 | 0.409560584 | -6.395643602 |
| VPS13A | 0.109917041 | -0.03317435 | 0.969114202 | 0.337001272 | 0.429912838 | -6.430788364 |
| PC | -0.156272674 | -0.03637411 | -0.959924199 | 0.341562341 | 0.43370474 | -6.439574185 |
| COL4A1 | 0.241449475 | 0.247754479 | 0.934889231 | 0.354192638 | 0.446608702 | -6.463098182 |
| NDUFC2 | 0.123675055 | 0.007584534 | 0.931828291 | 0.355757485 | 0.446608702 | -6.46593318 |
| MRPS14 | -0.13783758 | 0.04672819 | -0.930120341 | 0.35663259 | 0.446608702 | -6.467511152 |
| SLC25A26 | -0.110911683 | 0.020056667 | -0.913522413 | 0.365209494 | 0.455047652 | -6.482700115 |
| B3GALNT2 | -0.100621612 | 0.031397176 | -0.907579661 | 0.368312359 | 0.455047652 | -6.488074048 |
| NDUFA4 | -0.131899022 | 0.001032332 | -0.907465923 | 0.368371909 | 0.455047652 | -6.488176568 |
| HIF1A | -0.152257785 | 0.09419447 | -0.894073692 | 0.375426765 | 0.461673454 | -6.50016093 |
| TP53 | 0.112588087 | -0.067120309 | 0.856774469 | 0.395524425 | 0.484207032 | -6.532627788 |
| DTYMK | -0.160079581 | -0.0214155 | -0.848171352 | 0.400253317 | 0.487808731 | -6.539925604 |
| CPT2 | -0.116606271 | -0.028596716 | -0.83792309 | 0.405931934 | 0.492530747 | -6.548525482 |
| FKRP | 0.080236934 | -0.003551846 | 0.819417534 | 0.416310615 | 0.502888487 | -6.563796741 |
| RARS2 | 0.075887771 | 0.081627538 | 0.811276069 | 0.420927296 | 0.506225339 | -6.570410068 |
| LDHC | 0.17363971 | 0.143500379 | 0.804716543 | 0.424669331 | 0.508485647 | -6.575691562 |
| FOXRED1 | -0.112262484 | -0.021934366 | -0.777533357 | 0.440388522 | 0.525004658 | -6.597132571 |
| DAG1 | 0.150350689 | -0.122332954 | 0.745140882 | 0.459561486 | 0.545479503 | -6.621742378 |
| RMND1 | -0.085253728 | -0.089536534 | -0.709163914 | 0.481409411 | 0.568938395 | -6.647873846 |
| HMGCL | -0.059719156 | -0.013846212 | -0.695829557 | 0.489652549 | 0.576185973 | -6.657237141 |
| TMEM126B | 0.120021855 | 0.037242726 | 0.688284623 | 0.49435116 | 0.579218312 | -6.662457885 |
| COQ9 | -0.063627531 | 0.003845177 | -0.677399417 | 0.501173434 | 0.584702339 | -6.66989147 |
| PIEZO1 | -0.108793861 | -0.023917771 | -0.627591316 | 0.533034583 | 0.619227409 | -6.702419282 |
| TACO1 | -0.066192843 | 0.029627056 | -0.619682059 | 0.538189356 | 0.6225665 | -6.707359657 |
| MECR | 0.050665549 | -0.010411701 | 0.613564734 | 0.542193842 | 0.624552401 | -6.711138401 |
| TIMMDC1 | 0.051293445 | 0.008066998 | 0.606714865 | 0.546695943 | 0.62553365 | -6.715325801 |
| DNAJC19 | -0.061403786 | -0.023258571 | -0.602308755 | 0.549601913 | 0.62553365 | -6.717994814 |
| NDUFA10 | -0.053636344 | 0.040139302 | -0.597745692 | 0.552619637 | 0.62553365 | -6.720738683 |
| ISCU | 0.051140334 | 0.024293919 | 0.597354136 | 0.552878976 | 0.62553365 | -6.720973176 |
| SLC39A8 | 0.095262918 | 0.038145211 | 0.59490523 | 0.554502356 | 0.62553365 | -6.722436324 |
| MECP2 | 0.057641509 | 0.044804686 | 0.578338137 | 0.565547306 | 0.635367961 | -6.732178905 |
| TIMM22 | -0.049405314 | 0.005042953 | -0.574183688 | 0.568333962 | 0.635881851 | -6.73457942 |
| HS6ST2 | 0.116882759 | -0.184904752 | 0.562908108 | 0.575931103 | 0.641751801 | -6.741008523 |
| NSUN3 | 0.055109718 | -0.026216049 | 0.538431652 | 0.592590365 | 0.65763077 | -6.754530917 |
| TPK1 | -0.045012558 | 0.000276934 | -0.502059939 | 0.617757465 | 0.682784566 | -6.773526802 |
| HMGCS2 | 0.08163301 | -0.082851332 | 0.462902741 | 0.64537713 | 0.710435309 | -6.79250691 |
| NDUFS4 | 0.043346764 | 0.05529604 | 0.44219799 | 0.660190727 | 0.722653179 | -6.801925422 |
| CFH | -0.076423606 | -0.003776804 | -0.436680351 | 0.664161986 | 0.722653179 | -6.804363216 |
| ACADM | 0.051613049 | 0.024655302 | 0.436325956 | 0.664417392 | 0.722653179 | -6.804518756 |
| MTFMT | 0.038556755 | 0.05677557 | 0.431208699 | 0.668109756 | 0.723785569 | -6.806750689 |
| MRPS16 | 0.033250894 | 0.014710731 | 0.405175773 | 0.687020446 | 0.741330362 | -6.81770025 |
| ALDH6A1 | -0.040461569 | -0.019755097 | -0.34714788 | 0.729889734 | 0.783590344 | -6.839668026 |
| LDHAL6B | 0.145853197 | 0.130272075 | 0.344423755 | 0.731925047 | 0.783590344 | -6.840616448 |
| LDHD | -0.072913297 | -0.105728635 | -0.324895584 | 0.746571467 | 0.796148478 | -6.847197503 |
| SCO2 | 0.046698404 | 0.129750214 | 0.314581859 | 0.754345506 | 0.80130865 | -6.850518929 |
| COG8 | -0.039625441 | 0.130218908 | -0.272874127 | 0.786037237 | 0.827764245 | -6.862861552 |
| NFU1 | -0.029695452 | 0.047225022 | -0.269358987 | 0.788725821 | 0.827764245 | -6.863821961 |
| SLC25A13 | -0.030796493 | -0.035524817 | -0.266518331 | 0.790900415 | 0.827764245 | -6.864589015 |
| CARS2 | -0.028207162 | -0.033935336 | -0.26589345 | 0.791379004 | 0.827764245 | -6.864756662 |
| TARS2 | -0.04929695 | -0.044095546 | -0.255625012 | 0.799254926 | 0.832811431 | -6.86745532 |
| PNKD | 0.019733366 | -0.062804841 | 0.231106728 | 0.818144279 | 0.84681029 | -6.8734702 |
| NDUFV1 | 0.017959237 | 0.038604069 | 0.230137294 | 0.818893467 | 0.84681029 | -6.873695597 |
| UPB1 | -0.031274926 | -0.075575505 | -0.210414452 | 0.834171493 | 0.859354029 | -6.878075907 |
| LDHAL6A | -0.047877171 | 0.058857556 | -0.193307776 | 0.847475553 | 0.867311242 | -6.881558099 |
| SERAC1 | 0.021844254 | 0.003281536 | 0.192314493 | 0.848249457 | 0.867311242 | -6.881751238 |
| RB1 | -0.022408916 | 0.011840382 | -0.164422495 | 0.870039862 | 0.886271949 | -6.886768965 |
| POLG | -0.011039066 | 0.002960644 | -0.147951763 | 0.882956495 | 0.896085959 | -6.889363929 |
| NDUFA9 | -0.011513925 | -0.012971668 | -0.110280535 | 0.912613974 | 0.922754129 | -6.894271048 |
| MRPL44 | -0.008919799 | -0.005018873 | -0.059607678 | 0.952697971 | 0.959728952 | -6.898614377 |
| PFKFB2 | -0.007589052 | -0.030946622 | -0.052393107 | 0.958417431 | 0.961941025 | -6.899022091 |
| SLC5A12 | 0.009300816 | 0.147710795 | 0.041448649 | 0.967097924 | 0.967097924 | -6.899540321 |
