# Supplementary material for: A lactate metabolism-related gene signature to diagnose osteoarthritis based on machine learning combined with experimental validation
Source: Aging (Albany NY). 2024 Oct 16;16(20):13076–103. doi: 10.18632/aging.205873 (PMC11552637; doi:10.18632/aging.205873)
Supplement: Supplementary Tables 7-11 [file aging-16-205873-s004.pdf]

Supplementary Table 7. The coefficients of the genes in the LASSO regression model.

Gene	Coefficients
XK	-0.466813114
GATA1	-0.297177278
HMOX1	-1.044903664
SLC2A1	-0.306222582
CYC1	-0.009444988
HAGH	-0.09719996
NDUFB9	-3.705789774
HTRA2	-0.00717518
ISCA1	-0.870643408
HIBCH	0.211640847
FKTN	0.211721146
PDP1	3.359955862
SLC16A8	0.103277647

Supplementary Table 8. The genes identified by the SVM-RFE algorithm.

Gene
NDUFB9, CYC1, HMOX1, PYGL, CFI, FKTN, MLIP, SLC2A1, CYP27A1, CLPB, NDUFS2, RHAG, HIBCH.

Supplementary Table 9. The results obtained from the Boruta algorithm.

Gene	Status
RHAG	Confirmed
XK	Confirmed
GATA1	Confirmed
SLC4A1	Confirmed
RHCE	Confirmed
KLF1	Confirmed
RHD	Confirmed
HMOX1	Confirmed
LDHA	Confirmed
EMB	Confirmed
SLC2A1	Confirmed
PYGL	Confirmed
CLPB	Confirmed
CYC1	Confirmed
C1QBP	Confirmed
HAGH	Confirmed
MYC	Confirmed
LDHB	Confirmed
NDUFB9	Confirmed
NDUFS2	Confirmed
MRPL12	Confirmed
ALDOA	Confirmed

COX8A	Confirmed
COQ2	Confirmed
NDUFV2	Confirmed
HTRA2	Confirmed
CHCHD10	Confirmed
MDH2	Confirmed
NDUFAF2	Confirmed
NDUFAF3	Confirmed
TUFM	Confirmed
TIMM50	Confirmed
PDHX	Confirmed
MTHFD1	Confirmed
SLC25A12	Confirmed
CYP27A1	Confirmed
ADAMTS13	Confirmed
POMT1	Confirmed
HIBCH	Confirmed
FKTN	Confirmed
PDP1	Confirmed
CFI	Confirmed
MLIP	Confirmed
SLC16A8	Confirmed
PNPLA2	Rejected
HSD17B10	Rejected
JAK2	Rejected
SLC5A8	Rejected
LONP1	Rejected
MRPL3	Rejected
NDUFAF4	Rejected
PNPLA8	Rejected
SUCLG1	Rejected
CAV3	Rejected
PER2	Rejected
SPP1	Rejected
ABCG8	Rejected
GYS2	Tentative
HBB	Tentative
SDHB	Tentative
UQCRH	Tentative
NDUFA6	Tentative
COX5A	Tentative
PUS1	Tentative
RNASEH1	Tentative
MRPS7	Tentative
SLC16A1	Tentative
COX10	Tentative
HPDL	Tentative
ISCA1	Tentative
PIGA	Tentative
COX4I1	Tentative
PLEC	Tentative
GATA2	Tentative

Supplementary Table 10. The univariate logistic regression of the differentially-expressed genes associated with lactate metabolism.

Gene	OR (95%CI)	P-value
SLC2A1	0.037 (0.004-0.173)	<0.001
SLC25A12	143.349 (11.744-4357.33)	0.001
HIBCH	1172.193 (41.919-208455.093)	0.001
LDHB	0.021 (0.001-0.140)	0.001
COQ2	0.003 (0.001-0.054)	0.001
TIMM50	0.002 (0.001-0.047)	0.001
COX8A	0.009 (0.001-0.099)	0.001
MTHFD1	0.004 (0.001-0.068)	0.001
SLC16A8	20.916 (4.512-199.328)	0.001
C1QBP	0.021 (0.001-0.145)	0.001
MDH2	0.001 (0.001-0.037)	0.001
MRPL12	0.017 (0.001-0.135)	0.001
MYC	0.038 (0.003-0.207)	0.002
PLEC	37.541 (5.505-573.250)	0.002
NDUFA6	0.010 (0.001-0.113)	0.002
CYP27A1	258.300 (14.192-19613.825)	0.002
ISCA1	0.005 (0.001-0.089)	0.002
NDUFV2	0.003 (0.001-0.064)	0.002
TUFM	0.001 (0.001-0.021)	0.002
NDUFAF3	0.001 (0.001-0.012)	0.002
SUCLG1	0.013 (0.001-0.147)	0.002
RHAG	0.535 (0.324-0.747)	0.002
HBB	0.241 (0.081-0.538)	0.002
GATA2	10.556 (2.938-69.825)	0.002
CHCHD10	0.025 (0.001-0.190)	0.002
GYS2	0.226 (0.073-0.523)	0.003
PDHX	0.053 (0.006-0.304)	0.003
XK	0.450 (0.236-0.697)	0.003
EMB	0.190 (0.051-0.477)	0.003
ADAMTS13	42.235 (5.112-805.013)	0.003
HAGH	0.106 (0.017-0.375)	0.003
ALDOA	0.003 (0.001-0.063)	0.003
NDUFS2	0.001 (0.001-0.014)	0.003
UQCRH	0.018 (0.001-0.166)	0.003
NDUFB9	0.001 (0.001-0.004)	0.003
SLC4A1	0.293 (0.105-0.565)	0.003
LDHA	0.142 (0.028-0.406)	0.003
HPDL	0.018 (0.001-0.184)	0.003
SDHB	0.013 (0.001-0.135)	0.003
RNASEH1	0.005 (0.001-0.098)	0.004
MLIP	345.260 (17.277-80337.860)	0.004
CLPB	0.001 (0.001-0.039)	0.004
GATA1	0.435 (0.214-0.689)	0.004
PUS1	0.039 (0.003-0.252)	0.004
PYGL	0.009 (0.001-0.116)	0.004
COX4I1	0.021 (0.001-0.206)	0.004

MRPS7	0.002 (0.001-0.063)	0.004
HSD17B10	0.029 (0.001-0.209)	0.004
COX10	0.003 (0.001-0.090)	0.004
PIGA	0.050 (0.004-0.311)	0.005
HTRA2	0.001 (0.001-0.009)	0.005
PDP1	181.034 (10.849-17070.709)	0.005
COX5A	0.020 (0.001-0.188)	0.005
KLF1	0.451 (0.224-0.728)	0.005
JAK2	0.069 (0.007-0.348)	0.006
NDUFAF4	0.113 (0.020-0.481)	0.006
PNPLA8	0.063 (0.005-0.353)	0.006
NDUFAF2	0.112 (0.017-0.464)	0.007
RHD	0.514 (0.279-0.791)	0.008
FKTN	119875.058 (245.767-44103618194.23)	0.009
LONP1	0.051 (0.003-0.341)	0.010
ABCG8	10.071 (2.296-80.359)	0.010
SLC5A8	0.190 (0.045-0.611)	0.010
SLC16A1	0.126 (0.021-0.532)	0.010
MRPL3	0.146 (0.028-0.567)	0.010
SPP1	3.812 (1.445-12.166)	0.012
PER2	5.184 (1.569-22.371)	0.012
PNPLA2	0.147 (0.023-0.521)	0.013
RHCE	0.688 (0.492-0.913)	0.014
POMT1	15336.767 (60.167-493515289.745)	0.014
CAV3	10.665 (2.035-102.238)	0.014
HMOX1	0.001 (0.001-0.021)	0.017
CFI	708510.387 (272.556-4445894389564.95)	0.017
CYC1	0.001 (0.000-0.001)	0.217

Supplementary Table 11. The samples in the C1 and C2 clusters.

Cluster	Sample ID
C1	GSM1248759, GSM1248760, GSM1248761, GSM1248762, GSM1248764, GSM1248765, GSM1248766, GSM1248767.
C2	GSM1248763, GSM1248768, GSM1248769, GSM1248770, GSM1248771, GSM1248772, GSM1248773, GSM1248774, GSM1248775, GSM1248776, GSM1248777, GSM1248778, GSM1248779, GSM1248780, GSM1248781, GSM1248782, GSM1248783, GSM1248784, GSM1248785, GSM1248786, GSM1248787, GSM1248788, GSM1248789, GSM1248790, GSM1248791, GSM1248792, GSM1248793, GSM1248794, GSM1248795, GSM1248796, GSM1248797, GSM1248798, GSM1248799, GSM1248800, GSM1248801, GSM1248802, GSM1248803, GSM1248804, GSM1248805, GSM1248806, GSM1248807, GSM1248808.
