# Supplementary material for: A lactate metabolism-related gene signature to diagnose osteoarthritis based on machine learning combined with experimental validation
Source: Aging (Albany NY). 2024 Oct 16;16(20):13076–103. doi: 10.18632/aging.205873 (PMC11552637; doi:10.18632/aging.205873)
Supplement: Supplementary Table 12 [file aging-16-205873-s005.docx]

Supplementary Table 12. The differentially-expressed genes between C1 and C2 subjects.

| **Gene Symbol** | **logFC** | **AveExpr** | **t** | **P value** | **FDR** | **B** |
| --- | --- | --- | --- | --- | --- | --- |
| SLC7A5 | -2.938837679 | 0.310734612 | -12.00258297 | 1.58E-16 | 1.55E-12 | 27.22262473 |
| HECW2 | 2.134174999 | -0.174919201 | 11.90845967 | 2.14E-16 | 1.55E-12 | 26.9356857 |
| CRISP3 | -2.970080491 | 0.372230413 | -11.03299203 | 3.66E-15 | 9.96E-12 | 24.20753483 |
| TLR7 | 2.680207394 | -0.262266064 | 10.9168452 | 5.38E-15 | 1.30E-11 | 23.83764542 |
| FPR1 | -3.593335189 | 0.650903529 | -9.972914199 | 1.30E-13 | 1.22E-10 | 20.76493578 |
| CA2 | -3.326464234 | 0.249391369 | -9.778945304 | 2.53E-13 | 1.97E-10 | 20.11938545 |
| DHRS13 | -2.182249377 | 0.326687686 | -9.711588358 | 3.20E-13 | 2.24E-10 | 19.89414024 |
| MED12L | -2.070617627 | 0.047033155 | -9.67032936 | 3.69E-13 | 2.36E-10 | 19.7558988 |
| KIAA1377 | 2.177006247 | -0.236842954 | 9.614229566 | 4.48E-13 | 2.70E-10 | 19.56760705 |
| SLC11A1 | -2.19877368 | 0.227626874 | -9.512263185 | 6.38E-13 | 3.75E-10 | 19.22442165 |
| TRH | -2.202747646 | 0.04073726 | -9.263099053 | 1.53E-12 | 6.92E-10 | 18.3807996 |
| FOXS1 | 3.26702741 | -0.347560051 | 8.825634586 | 7.14E-12 | 2.55E-09 | 16.88338731 |
| SLC25A37 | -2.669578842 | 0.288105782 | -8.741731076 | 9.63E-12 | 3.22E-09 | 16.59398431 |
| IL1R2 | -3.816226836 | 0.466199683 | -8.589135861 | 1.66E-11 | 4.71E-09 | 16.06595647 |
| GADD45A | -2.339588107 | 0.348196791 | -8.449037531 | 2.74E-11 | 7.17E-09 | 15.57934197 |
| ORM1 | -2.540092069 | 0.194880512 | -8.344504765 | 3.98E-11 | 9.62E-09 | 15.21518301 |
| GYPA | -2.996499668 | 0.633559184 | -8.304074115 | 4.61E-11 | 1.07E-08 | 15.07409972 |
| LOC100128420 | 2.220684064 | -0.057241086 | 8.183508374 | 7.11E-11 | 1.45E-08 | 14.6526393 |
| SLC25A21 | -2.101698935 | 0.18614352 | -8.039548965 | 1.19E-10 | 2.17E-08 | 14.14802642 |
| HTR2B | 2.331634944 | -0.138220697 | 7.844418053 | 2.42E-10 | 3.84E-08 | 13.46189283 |
| RAB7B | 2.426757381 | -0.303005143 | 7.748759735 | 3.43E-10 | 4.93E-08 | 13.12472828 |
| RASSF9 | 2.660194662 | -0.272931484 | 7.697908446 | 4.12E-10 | 5.74E-08 | 12.94530118 |
| AREG | -3.712958102 | 0.249786218 | -7.613859716 | 5.59E-10 | 7.37E-08 | 12.64846881 |
| IRGM | -2.086732268 | -0.062312377 | -7.505414002 | 8.30E-10 | 1.02E-07 | 12.26502765 |
| C5AR1 | -2.382837625 | 0.195747007 | -7.479296945 | 9.12E-10 | 1.11E-07 | 12.17261567 |
| HBG1 | -4.543547955 | 0.411573243 | -7.466837275 | 9.55E-10 | 1.15E-07 | 12.12852014 |
| LRRC7 | 2.10909248 | -0.023175853 | 7.44662688 | 1.03E-09 | 1.19E-07 | 12.05698302 |
| ADM | -3.001254776 | 0.182286177 | -7.441612801 | 1.05E-09 | 1.20E-07 | 12.03923298 |
| ALB | -2.886073171 | 0.243957659 | -7.300961339 | 1.75E-09 | 1.82E-07 | 11.54101434 |
| SNCA | -2.452757329 | 0.214099072 | -7.160854772 | 2.91E-09 | 2.74E-07 | 11.04426096 |
| HBQ1 | -3.4448007 | 0.353464637 | -7.159974906 | 2.92E-09 | 2.74E-07 | 11.04114029 |
| FZD8 | 2.080627754 | -0.298416392 | 7.021207414 | 4.85E-09 | 3.93E-07 | 10.54886319 |
| HMBS | -2.369562486 | 0.31362681 | -7.01629472 | 4.94E-09 | 3.96E-07 | 10.53143294 |
| ARG1 | -4.408214389 | 0.763585407 | -6.934447412 | 6.66E-09 | 4.98E-07 | 10.24103327 |
| ALAS2 | -4.249667508 | 0.314583243 | -6.919023743 | 7.04E-09 | 5.14E-07 | 10.18630996 |
| PLIN2 | -2.767469796 | 0.359977233 | -6.88106053 | 8.09E-09 | 5.64E-07 | 10.05162093 |
| OR2G6 | -2.094500412 | -0.077016783 | -6.833204836 | 9.64E-09 | 6.49E-07 | 9.881849705 |
| CX3CR1 | 2.506985267 | -0.305366434 | 6.784991245 | 1.15E-08 | 7.42E-07 | 9.710834276 |
| PRRT2 | 2.056182405 | -0.143363017 | 6.772623332 | 1.20E-08 | 7.69E-07 | 9.666970049 |
| SLC22A4 | -2.618433069 | 0.380349322 | -6.736293813 | 1.37E-08 | 8.41E-07 | 9.538137944 |
| GABRB2 | 2.335498107 | -0.214788069 | 6.653460211 | 1.86E-08 | 1.06E-06 | 9.244492999 |
| F2RL3 | -2.39704789 | 0.395401726 | -6.641649169 | 1.94E-08 | 1.09E-06 | 9.202636345 |
| SLC4A1 | -2.607050365 | 0.189519787 | -6.628253946 | 2.04E-08 | 1.13E-06 | 9.155170171 |
| BEX1 | -2.245132687 | 0.071661308 | -6.609008114 | 2.18E-08 | 1.20E-06 | 9.086981236 |
| GLUL | -2.232876338 | 0.22434742 | -6.519496516 | 3.03E-08 | 1.53E-06 | 8.769993755 |
| NFIL3 | -2.287333505 | 0.220445271 | -6.467303602 | 3.66E-08 | 1.78E-06 | 8.585299872 |
| MEGF10 | 2.054034196 | -0.220846928 | 6.437118903 | 4.09E-08 | 1.94E-06 | 8.478538574 |
| DNTT | -3.738818574 | 0.755757014 | -6.420872707 | 4.34E-08 | 2.03E-06 | 8.421093973 |
| CXCL11 | 2.138378013 | -0.040482994 | 6.385263975 | 4.94E-08 | 2.25E-06 | 8.295230094 |
| IFIT1B | -4.397487987 | 0.694767621 | -6.348859393 | 5.64E-08 | 2.47E-06 | 8.16661983 |
| SRGN | -2.870223936 | 0.489705556 | -6.339831754 | 5.83E-08 | 2.53E-06 | 8.134737914 |
| LOC100129940 | 2.302861367 | -0.192859301 | 6.312809621 | 6.43E-08 | 2.69E-06 | 8.039334031 |
| IFIT1 | 2.082687107 | -0.004402056 | 6.296294705 | 6.83E-08 | 2.83E-06 | 7.98104745 |
| FLRT3 | 2.455335268 | 0.069880825 | 6.294386604 | 6.87E-08 | 2.83E-06 | 7.97431417 |
| FCAR | -2.283321287 | 0.201570592 | -6.132056787 | 1.24E-07 | 4.54E-06 | 7.402356139 |
| NAMPT | -2.128042884 | 0.268246792 | -6.108233194 | 1.35E-07 | 4.86E-06 | 7.318574251 |
| TMOD1 | -2.16709596 | -0.218929232 | -6.102138344 | 1.38E-07 | 4.96E-06 | 7.297147268 |
| SLC2A14 | -2.005487523 | 0.417678243 | -6.080554695 | 1.49E-07 | 5.26E-06 | 7.221292012 |
| HEMGN | -3.746815036 | 0.344190812 | -6.079836814 | 1.50E-07 | 5.26E-06 | 7.218769686 |
| PNMAL1 | 2.63821233 | 0.018521904 | 6.034793017 | 1.76E-07 | 6.02E-06 | 7.06059133 |
| PTX3 | -3.027079259 | 0.212501872 | -6.019961178 | 1.86E-07 | 6.25E-06 | 7.00854514 |
| RBM38 | -2.161726566 | 0.177632773 | -5.981001034 | 2.14E-07 | 6.91E-06 | 6.871924478 |
| EREG | -2.234178188 | 0.189081711 | -5.948086306 | 2.41E-07 | 7.51E-06 | 6.756612836 |
| SIGLEC12 | -2.424454457 | 0.039594431 | -5.868675459 | 3.21E-07 | 9.43E-06 | 6.478848266 |
| S100A9 | -4.717991758 | 1.006179011 | -5.838320585 | 3.59E-07 | 1.03E-05 | 6.372845564 |
| EMX2OS | 2.742932946 | -0.17918759 | 5.767069894 | 4.64E-07 | 1.26E-05 | 6.124431532 |
| PRG3 | -3.299588919 | 0.542909578 | -5.765002851 | 4.67E-07 | 1.27E-05 | 6.117233549 |
| CA1 | -4.481548374 | 0.715277066 | -5.742020151 | 5.07E-07 | 1.35E-05 | 6.037235991 |
| HBD | -3.523496298 | -0.107548665 | -5.678417191 | 6.37E-07 | 1.63E-05 | 5.816185775 |
| SLCO4A1 | -2.159695485 | 0.489940246 | -5.651325801 | 7.02E-07 | 1.76E-05 | 5.722186616 |
| CD5L | -2.751291983 | 0.240600847 | -5.614399025 | 8.02E-07 | 1.95E-05 | 5.594218056 |
| AHSP | -4.367322341 | 0.709073332 | -5.600120094 | 8.44E-07 | 2.02E-05 | 5.544784479 |
| CCL23 | -2.214482096 | 0.069899298 | -5.559508793 | 9.76E-07 | 2.27E-05 | 5.404343936 |
| CLEC4GP1 | 2.397168231 | -0.010655314 | 5.524550802 | 1.11E-06 | 2.52E-05 | 5.283642875 |
| OSBP2 | -2.175304445 | 0.117576106 | -5.511031691 | 1.16E-06 | 2.61E-05 | 5.237013064 |
| ADAMTS4 | -2.298223219 | 0.355028958 | -5.505116594 | 1.19E-06 | 2.65E-05 | 5.216619442 |
| EPB42 | -3.404399004 | 0.549409615 | -5.498463813 | 1.21E-06 | 2.69E-05 | 5.193688792 |
| CEACAM6 | -2.724398796 | 0.331456314 | -5.484749095 | 1.27E-06 | 2.81E-05 | 5.146438433 |
| OLFM4 | -4.119784769 | -0.208866843 | -5.459718682 | 1.39E-06 | 3.01E-05 | 5.060277511 |
| HBA2 | -3.412452896 | -0.007379847 | -5.440088174 | 1.49E-06 | 3.19E-05 | 4.992772984 |
| KLF1 | -2.456440307 | 0.340095626 | -5.39934531 | 1.73E-06 | 3.59E-05 | 4.852865287 |
| PRG2 | -3.895170368 | 0.880085724 | -5.356967112 | 2.01E-06 | 4.08E-05 | 4.707632321 |
| CEACAM8 | -4.657975012 | 0.885239028 | -5.336089538 | 2.16E-06 | 4.33E-05 | 4.636195413 |
| PRRT4 | -2.39790376 | -0.056774809 | -5.303237954 | 2.43E-06 | 4.73E-05 | 4.523940492 |
| TTTY16 | -2.021027624 | 0.239526922 | -5.28872184 | 2.56E-06 | 4.91E-05 | 4.474399354 |
| LINC00313 | -2.452962311 | 0.405305745 | -5.288130245 | 2.56E-06 | 4.91E-05 | 4.472381137 |
| HBM | -4.389431092 | 0.717369059 | -5.271180527 | 2.72E-06 | 5.15E-05 | 4.414584341 |
| RHAG | -4.080326276 | 0.677685599 | -5.220402302 | 3.25E-06 | 5.98E-05 | 4.24175226 |
| S100A8 | -4.656963214 | 0.857605388 | -5.211790322 | 3.35E-06 | 6.14E-05 | 4.212487945 |
| S100A12 | -5.060145971 | 1.103930722 | -5.174351536 | 3.83E-06 | 6.89E-05 | 4.085433153 |
| LALBA | -2.598367763 | 0.073560177 | -5.173006748 | 3.84E-06 | 6.91E-05 | 4.08087446 |
| LTF | -3.815792204 | 0.850166536 | -5.151463637 | 4.15E-06 | 7.31E-05 | 4.007894226 |
| S100P | -4.015843752 | 1.014046699 | -5.149071595 | 4.18E-06 | 7.35E-05 | 3.99979654 |
| DEFA3 | -5.87234766 | 0.445532528 | -5.097398541 | 5.01E-06 | 8.49E-05 | 3.825152415 |
| GATA1 | -2.805337854 | 0.451733768 | -5.073501941 | 5.45E-06 | 9.05E-05 | 3.744573004 |
| TCN1 | -3.438353393 | 0.810475643 | -5.020164306 | 6.57E-06 | 0.000104893 | 3.565155995 |
| LCN2 | -5.134026602 | 0.365894598 | -5.018264147 | 6.61E-06 | 0.000105468 | 3.558775601 |
| CDH1 | -2.32990332 | 0.258117816 | -4.996347848 | 7.13E-06 | 0.000112202 | 3.485242104 |
| TRIM58 | -2.670286271 | 0.450094739 | -4.977109299 | 7.63E-06 | 0.000118958 | 3.420781058 |
| EPX | -3.535576321 | 0.436368039 | -4.976976911 | 7.63E-06 | 0.000118958 | 3.420337767 |
| CXCL10 | 2.351708637 | -0.292826642 | 4.958173117 | 8.15E-06 | 0.000125559 | 3.357414644 |
| NFE2 | -3.927748828 | 0.764429066 | -4.946441666 | 8.49E-06 | 0.000129347 | 3.318198551 |
| AQP9 | -2.154571553 | 0.286035508 | -4.932412862 | 8.91E-06 | 0.00013427 | 3.271344445 |
| CLEC1B | -3.052847638 | 0.317417907 | -4.925265099 | 9.14E-06 | 0.000136696 | 3.247489552 |
| BPI | -3.5528176 | 0.576178182 | -4.921887101 | 9.24E-06 | 0.000137833 | 3.236219993 |
| THY1 | 2.379442228 | 0.232499595 | 4.914119696 | 9.50E-06 | 0.000141017 | 3.210316786 |
| SLC16A10 | -2.083678891 | -0.04940652 | -4.867188942 | 1.12E-05 | 0.000160318 | 3.054113829 |
| LRRC17 | 2.03770763 | -0.033263719 | 4.858437987 | 1.15E-05 | 0.000163943 | 3.025046018 |
| EIF1AY | -2.46144556 | -0.994760345 | -4.846021208 | 1.20E-05 | 0.000169465 | 2.983833616 |
| CYP4F3 | -3.546837489 | 0.604225797 | -4.817062144 | 1.33E-05 | 0.000183698 | 2.88786373 |
| RNASE2 | -3.863658234 | 0.681312252 | -4.810292898 | 1.36E-05 | 0.000187438 | 2.865460704 |
| XK | -2.756465252 | 0.43251065 | -4.773000604 | 1.55E-05 | 0.000208739 | 2.742248732 |
| ALOX12 | -2.341641499 | 0.123626269 | -4.767771041 | 1.57E-05 | 0.000211343 | 2.724998944 |
| LIN7A | -2.066864381 | 0.085718932 | -4.758332042 | 1.63E-05 | 0.000216039 | 2.693882182 |
| ADD2 | -2.475020848 | 0.240015311 | -4.736291958 | 1.75E-05 | 0.000228542 | 2.621314861 |
| MMP1 | 3.006320283 | 0.533773975 | 4.733924213 | 1.77E-05 | 0.000229853 | 2.613526606 |
| CEACAM3 | -2.806919922 | 0.265766948 | -4.69021626 | 2.05E-05 | 0.000260106 | 2.470025408 |
| RGS1 | -2.177907536 | 0.001731918 | -4.669774478 | 2.20E-05 | 0.00027618 | 2.403088151 |
| C19orf59 | -2.054749769 | 0.140568281 | -4.663578621 | 2.25E-05 | 0.000280786 | 2.382822201 |
| PGLYRP1 | -3.500833005 | 0.880108142 | -4.646525168 | 2.38E-05 | 0.000294383 | 2.327096981 |
| FGG | -2.063265125 | -0.077899293 | -4.640660269 | 2.43E-05 | 0.000299813 | 2.30795103 |
| PADI4 | -3.547428396 | 0.695377347 | -4.618277925 | 2.62E-05 | 0.000319199 | 2.234972446 |
| GYPB | -2.787923888 | 0.324096822 | -4.58819338 | 2.91E-05 | 0.000345803 | 2.137104126 |
| ANK1 | -2.265001763 | 0.393073879 | -4.586589852 | 2.92E-05 | 0.000347309 | 2.131894948 |
| CYS1 | 2.248418391 | -0.190337268 | 4.575803152 | 3.03E-05 | 0.0003579 | 2.096872869 |
| DEFA4 | -5.075499546 | 0.993501819 | -4.525198801 | 3.60E-05 | 0.000411455 | 1.933025508 |
| VSTM1 | -3.083551508 | 0.457432841 | -4.513596176 | 3.74E-05 | 0.000425061 | 1.895565364 |
| PROK2 | -2.848924512 | 0.357620222 | -4.51296224 | 3.75E-05 | 0.000425539 | 1.893519804 |
| PPBP | -3.151185834 | 0.409076663 | -4.481065178 | 4.17E-05 | 0.000462941 | 1.790752692 |
| SLC36A2 | 2.137265366 | -0.150594595 | 4.476116607 | 4.24E-05 | 0.000469038 | 1.774836979 |
| RNASE3 | -3.742435403 | 0.759239437 | -4.460532049 | 4.47E-05 | 0.000489783 | 1.724762779 |
| KEL | -2.974516854 | 0.136054913 | -4.438941636 | 4.81E-05 | 0.000520044 | 1.655515815 |
| GLT1D1 | -2.176173595 | 0.439664115 | -4.376823734 | 5.92E-05 | 0.00061096 | 1.457106155 |
| AZU1 | -4.49322596 | 1.131578468 | -4.362216134 | 6.21E-05 | 0.000636614 | 1.410628498 |
| AMBP | -2.037775979 | 0.304971952 | -4.352494616 | 6.42E-05 | 0.000653253 | 1.379735737 |
| MPO | -4.913102848 | 1.299721633 | -4.314647327 | 7.28E-05 | 0.000725863 | 1.259763062 |
| FAM201A | -2.782453574 | 0.522775302 | -4.288799477 | 7.93E-05 | 0.000775837 | 1.178103092 |
| PF4V1 | -3.202267121 | 0.554943425 | -4.282169931 | 8.11E-05 | 0.000787419 | 1.157195141 |
| FAP | 2.153112098 | 0.156229628 | 4.266855715 | 8.53E-05 | 0.000819479 | 1.108955308 |
| ACSL6 | -2.832825997 | 0.468497184 | -4.262861255 | 8.64E-05 | 0.000828532 | 1.096385983 |
| TUBB1 | -2.353499513 | 0.456584703 | -4.238794446 | 9.35E-05 | 0.000885277 | 1.020772013 |
| CLC | -3.828519231 | 0.671903565 | -4.208975186 | 0.000103175 | 0.000959831 | 0.927365489 |
| PRTN3 | -4.134109315 | 1.111158331 | -4.162880723 | 0.000120007 | 0.001084858 | 0.783600045 |
| FCN1 | -2.013030058 | 0.173902105 | -4.151173068 | 0.00012469 | 0.001122057 | 0.747206758 |
| PF4 | -3.230032061 | 0.63338974 | -4.139637427 | 0.000129477 | 0.001155568 | 0.711397112 |
| CAMP | -4.606419713 | 0.633870516 | -4.124015121 | 0.000136245 | 0.001201177 | 0.662979335 |
| ELANE | -4.066270593 | 1.162118087 | -4.100363924 | 0.000147148 | 0.001280681 | 0.589849943 |
| COL3A1 | 2.63103142 | 0.329280093 | 4.075578397 | 0.00015948 | 0.00136883 | 0.513438017 |
| APCDD1L | 3.01870693 | 0.496064402 | 4.047573577 | 0.000174618 | 0.001474291 | 0.427381607 |
| MATN4 | 2.558273852 | 0.702779528 | 4.002992504 | 0.000201626 | 0.001649811 | 0.291011279 |
| RHD | -2.098792875 | 0.271941698 | -3.981692259 | 0.000215915 | 0.001737951 | 0.226129857 |
| CCL8 | 3.011866862 | 0.340242626 | 3.974209932 | 0.000221163 | 0.001771013 | 0.20338101 |
| HPR | -2.485849567 | 0.291607894 | -3.907150528 | 0.000274032 | 0.002097793 | 0.000501943 |
| CNTNAP2 | 2.120107404 | -0.291772193 | 3.883439347 | 0.000295494 | 0.002222954 | -0.070793722 |
| SERPINB10 | -2.158610219 | 0.394069322 | -3.84726336 | 0.00033139 | 0.002429931 | -0.179118052 |
| RETN | -2.952921305 | 0.329704301 | -3.809340802 | 0.000373514 | 0.002669484 | -0.292078484 |
| RHCE | -2.851862877 | 0.587710459 | -3.803665453 | 0.000380246 | 0.002703374 | -0.308930797 |
| STMN2 | 4.406485873 | 1.384124221 | 3.782743695 | 0.000406083 | 0.002846113 | -0.370935436 |
| C17orf99 | -2.874370737 | 0.505623628 | -3.743105447 | 0.000459733 | 0.003143102 | -0.487885444 |
| EGFL6 | 2.248466732 | 0.156042743 | 3.681232547 | 0.000557309 | 0.003642524 | -0.669042089 |
| DEFA8P | -2.634208964 | 0.738687282 | -3.64924557 | 0.000615249 | 0.003950425 | -0.762016134 |
| SMPD3 | 2.519099167 | -0.37031975 | 3.633311638 | 0.000646222 | 0.004105987 | -0.808154312 |
| VPREB1 | -2.821780734 | 0.549376601 | -3.601768544 | 0.000711994 | 0.004434103 | -0.899141653 |
| NOX5 | 2.009213421 | 0.301150115 | 3.60113053 | 0.000713388 | 0.004441513 | -0.900977219 |
| XIST | 5.313729884 | 2.194416878 | 3.592283222 | 0.000732992 | 0.004544051 | -0.926411125 |
| DDX3Y | -3.53713724 | -2.23823387 | -3.57867848 | 0.000764147 | 0.004690377 | -0.965449274 |
| FAM70A | 2.011248498 | -0.053836674 | 3.567022917 | 0.00079184 | 0.004827817 | -0.998824378 |
| SLC22A16 | -2.059158981 | 0.333272561 | -3.550192602 | 0.000833526 | 0.005037998 | -1.046902507 |
| E2F8 | -2.397004028 | 0.328987754 | -3.514807297 | 0.000928102 | 0.005475674 | -1.147539882 |
| HP | -2.376263667 | 0.231473067 | -3.508435653 | 0.000946184 | 0.005562735 | -1.165596423 |
| SLC26A8 | -2.019269141 | 0.275079242 | -3.490526739 | 0.000998826 | 0.005807828 | -1.216241718 |
| PRSS57 | -3.343296855 | 1.041180039 | -3.462603307 | 0.001086507 | 0.006199933 | -1.294891434 |
| UTY | -2.686934044 | -1.485252684 | -3.457845108 | 0.00110216 | 0.00627115 | -1.308254814 |
| RGL4 | -2.696596392 | 0.622915021 | -3.433995633 | 0.001183907 | 0.006630426 | -1.375065013 |
| REP15 | 2.073894671 | 0.318066974 | 3.424734642 | 0.001217178 | 0.006771408 | -1.400930869 |
| NCRNA00185 | -2.731285274 | -1.570109891 | -3.365488095 | 0.001451993 | 0.007823488 | -1.565371111 |
| RPS4Y1 | -5.04984198 | -3.790973272 | -3.338413299 | 0.001573061 | 0.008327225 | -1.639914444 |
| RPS4Y2 | -4.529319047 | -3.329620225 | -3.301101867 | 0.00175565 | 0.009075032 | -1.742011292 |
| CST7 | -2.23277865 | 0.28192509 | -3.258180017 | 0.001990466 | 0.010066229 | -1.858543918 |
| E2F2 | -2.201678716 | 0.568420503 | -3.256749615 | 0.00199878 | 0.010091845 | -1.862410396 |
| SPTA1 | -2.732208121 | 0.801473316 | -3.237652462 | 0.002112977 | 0.010565371 | -1.913925113 |
| CEBPE | -2.501393258 | 0.907302592 | -3.12508777 | 0.002921317 | 0.013661508 | -2.213484837 |
| TTTY15 | -2.904921618 | -1.905447056 | -3.115543138 | 0.003001816 | 0.013966671 | -2.238558375 |
| TXLNG2P | -2.405434869 | -1.585848408 | -3.11494878 | 0.003006898 | 0.013977541 | -2.240118025 |
| MS4A3 | -2.245984381 | 0.335706475 | -3.010796455 | 0.004033116 | 0.017703206 | -2.510274701 |
| CTSG | -2.98846968 | 0.626853024 | -2.956647191 | 0.004688032 | 0.01996132 | -2.648209484 |
| HIST2H3A | -2.219512175 | 0.448761019 | -2.940958064 | 0.004895547 | 0.020634252 | -2.68784569 |
| COL2A1 | 2.549209928 | -0.501782049 | 2.716699849 | 0.008962194 | 0.033249385 | -3.23770617 |
| IL11 | 2.180484799 | 0.895478496 | 2.709476434 | 0.009134266 | 0.033750522 | -3.254884336 |
| USP9Y | -2.10411045 | -1.40039772 | -2.634258692 | 0.011115161 | 0.039428083 | -3.431719448 |
